# A swab and wetting agent combination investigation for enhanced touch DNA recovery: A step towards standardisation

**DOI:** 10.1007/s00414-026-03907-w

**Published:** 2026-07-04

**Authors:** Hilary Arsenault, Roland A.H. van Oorschot, Duncan Taylor, Adrian Linacre, Mariya Goray

**Affiliations:** 1 College of Science and Engineering, Flinders University, Adelaide, Australia; 2 Office of the Chief Forensic Scientist, Victoria Police Forensic Services, Macleod, Australia; 3 School of Agriculture, Biomedicine and Environment, La Trobe University, Bundoora, Australia; 4 Forensic Science SA, Adelaide, Australia

**Keywords:** DNA recovery, Swab, Wetting agent, Casework, Touch DNA

## Abstract

Swabs are commonly used in forensic contexts to recover biological material from surfaces. Previous investigations have shown that there is no single best swab and wetting agent combination for DNA recovery. Touch DNA is often present in low quality and quantity, and the recovery and processing methods used during casework can result in DNA loss, therefore even small improvements in recovery could influence successful detection. Therefore, a DNA recovery method tailored for a touch DNA workflow is fundamental to effective forensic casework. In this study, combinations of five swabs (viscose, cotton, nylon, foam and polyester) and three wetting agents (water, 100% isopropanol (IPA) and 2% sodium dodecyl sulphate (SDS)) were used to collect samples of synthetically and naturally derived touch DNA from a polypropylene surface. Surfaces were swabbed using a single systematic swabbing method and extracted using the DNA IQ™ system. Interactions between the swabs and wetting agents had the greatest effect on DNA recovery, and polyester swabs moistened with SDS were among the combinations with the highest DNA yield under these experimental conditions. The worst-performing swabs differed by DNA type sampled. Polyester, viscose and cotton swabs moistened with water were amongst the combinations yielding the lowest DNA recovery for the synthetic touch deposits, whereas foam and nylon swabs moistened with IPA had the lowest yield for natural touch deposits. This research aims to contribute towards the development of a standardised touch DNA recovery method applicable to the sampling of polypropylene surfaces and other relevant touch DNA collection related objectives, such as investigations into DNA transfer and shedder testing.

**Supplementary Information:**

The online version contains supplementary material available at https://doi.org/10.1007/s00414-026-03907-w.

## Introduction

Trace DNA samples collected from objects believed to have been handled or touched at a crime scene are the most common type of DNA evidence submitted for analysis in forensic laboratories [1, 2]. Swabbing is a common and widely used method for recovering biological materials from items of forensic interest [2–4]. Recent investigations aimed at evaluating and improving the use of swabs for biological material collection have revealed that recovery efficiency is influenced by several variables. The main variables include: swab material [2, 3, 5–11], wetting agent [6, 7, 9, 11–15], surface characteristics [3, 6–8, 13], biological material [3, 11, 13], and the extraction process [7, 15–18].

The swab type has been identified as a variable that significantly affects DNA recovery from a surface [2, 3, 7, 11, 19, 20]. Many different types of swabs are commercially available; however, commonly used forensic casework swabs are often composed of cotton, foam, nylon, rayon (viscose), or polyester [3, 9, 20]. Therefore, these types of swabs have been extensively investigated to determine the most effective type for maximising DNA recovery; this body of literature was recently reviewed by Conte et al. [20]. Previous work found that the material from which the swab head is made can influence the ability of the swab to collect DNA from a surface [2, 3, 6–10] and affect the efficiency of DNA release during extraction [3, 5, 8, 10, 11]. The ability of the swab to collect and release DNA is, in part, attributed to the different types of bonds formed between DNA and the various swab head materials [3, 21, 22]. For example, swab material that forms hydrogen bonds with cell membranes and cell free DNA (cfDNA) during collection from a surface results in strong bonds that may allow for better DNA pick up but are difficult to break during the extraction process [21]. On the other hand, materials that form weaker dipole-dipole interactions allow better release of DNA from a swab during DNA extraction [21]. However, the material type and the bonds created with biological material alone cannot fully define the efficiency of certain swabs, as differences in DNA recovery efficiency among swabs made with the same material but produced by different manufacturers have been identified [3, 7, 8, 10].

These investigations also demonstrate that swabs perform differently depending on the type of biological material collected [3, 9, 11]. The recovery efficiency of DNA has been shown to vary, with blood, saliva, and touch DNA samples yielding different recovery rates when collected with the same swab under the same conditions [3, 9]. According to Verdon et al. [3], who directly compare recovery differences amongst biological materials from a smooth, non-porous surface, cotton swabs provided the highest recovery from blood and saliva, and polyester swabs provided the highest recovery for touch DNA deposits. However, other studies that do not directly compare biological materials indicate that saliva is best recovered with cotton [6, 11, 16], blood with cotton and foam [7, 11], and touch DNA with cotton, viscose, and nylon floq swabs [2, 7, 8, 10, 11, 23].

Similarly, swabs have been shown to perform differently depending on the surface characteristics of the substrate being swabbed [2, 3, 6, 7, 11]. Differences in DNA yield from non-porous and porous surfaces have been reported, even when collection was performed under the same experimental conditions [2, 3, 6, 7, 11]. For instance, Verdon et al. [3] found that for maximising touch DNA recovery on a smooth non-porous substrate, a polyester swab produced the highest yield. However, when swabbing a rough, porous substrate or a rough, non-porous substrate, a foam or cotton swab, respectively, produced the highest yields [3]. It is hypothesised that the interaction between a swab and a porous surface affects successful recovery, with flexible swabs, such as foam, performing better because they can penetrate porous surfaces more effectively than less flexible swabs [3].

Further, swab related variables such as the size and fibre coil of a swab head [3, 21, 24], swab absorption capabilities [3, 11, 21, 24], swabbing technique [6, 25–29], swab storage [3], ease of use of a swab [6], and the skill of the practitioner [6], have also been shown to impact DNA recovery. The arrangement of the swab head material around the swab shaft can influence DNA recovery [3, 21]. Tightly wound swabs can decrease DNA collection and subsequently trap the collected DNA, thereby creating a physical barrier that decreases DNA release during extraction [21, 24]. It has also been indicated that the absorption capacity of the swab influences DNA recovery and extraction efficiency, such that larger, loosely bound swabs tend to have a higher absorption efficiency, meaning they can collect more DNA from a surface and release it more efficiently during extraction [3, 10, 11, 16, 21, 24]. The swabbing technique has also been associated with increased recovery, with double swabbing, swab rotation, swab pressure, swab angle, and multiple passes over the target area shown to enhance DNA recovery [6, 26, 28–30]. Additionally, the method of storing the swab after sampling has been highlighted as a potential source of DNA loss due to degradation after collection. Prolonged exposure of the swab to moisture can increase DNA degradation; therefore, swab containers with built-in ventilation systems, preservatives, or storage at −20 °C have been shown to yield higher DNA amounts [2, 3, 31, 32]. Additionally, the efficiency of collecting DNA from a surface has been attributed to the skill of the practitioner and the ease of use of the swab, with skilled practitioners and swabs that are easiest to handle tending to produce higher DNA yields [6, 8, 11]. Often, the swab type has been found to significantly influence DNA recovery; however, likely due to other experimental factors, no single swab type has proven superior for touch DNA recovery across all touch DNA deposits and variables [2, 7].

Both the type [7, 9, 12–15] and volume [6, 11, 12] of the wetting agent used to moisten a swab have also been identified as important variables, capable of impacting DNA recovery from a surface. Water is commonly used to moisten swabs, likely because it is readily available and low-cost [9, 12]. However, many studies have shown that the use of alternative solutions can increase DNA recovery over the use of water [7, 9, 12–15]. Detergents, such as sodium dodecyl sulphate (SDS) [7, 12–15], Triton X 100 [12–14, 33] and Tween 20 [13, 14], alcohols, such as isopropanol [5, 7, 9, 34] and ethanol [7, 9, 12], and isotonic solutions, such as phosphate-buffered saline (PBS) [7, 12] and NaCl [15], have all been investigated as possible alternative wetting agents to increase DNA recovery. Unlike with swabs, the literature shows clear trends that suggest that using a detergent, specifically SDS [12–14], or an alcohol (70% −100% isopropanol (IPA) or ethanol (EtOH)) [5, 9, 34] to moisten swabs prior to DNA collection can improve recovery over water moistened swabs. This is likely due to SDS’s ability to solubilise cellular components, thereby enhancing cellular recovery, and to alcohol’s ability to weaken the electrostatic effects and cellular membranes and to disrupt hydrophobic interactions, destabilising surface adhesion to substrates, making DNA more readily available for recovery [14, 35, 36]. Yet, while there has been some movement in the introduction of isopropanol into casework [5, 9, 34], no such shift has been seen in the implementation of SDS, despite numerous publications reporting increased DNA yield when swabbing was performed with this solution compared with several other wetting agents [7, 12–14].

As seen with swabs, the type of biological material being collected also impacts the ability of wetting agents to recover DNA from a surface. Wetting agents, such as detergents and water, have been identified to maximise DNA recovery when collecting cellular and cfDNA, known components of touch DNA samples, and natural touch deposits [7, 12–14, 37, 38]. Alternatively, chelating agents and alcohols have been identified as the most suitable for collecting body fluids, such as blood and saliva [9, 13, 19].

The ability of a wetting agent to increase DNA yield has also been shown to depend on surface characteristics, as observed with swabs [7, 13]. Different volumes of a wetting agent can have different interactions with a surface; for instance, DNA yield from porous items has been shown to significantly increase when larger volumes of wetting agents are applied to a swab, whereas on non-porous items, volume seems to have less of an impact [6, 11, 12].

Other factors, such as the extraction kit [15–17], platform [16], and protocol [7, 16–18] used to extract DNA from a swab influences extraction efficiency (the ability of DNA to be released from the swab), which, in turn, affects the overall recovery efficiency. Of the DNA present on a swab, 15–94% [10, 18, 39, 40] may remain unrecovered and lost during the extraction process. When considering both collection and extraction, the total amount of unrecovered and lost DNA can range from 20 to 98% of the initial deposit; however, it is typically around 50–75% [5, 7, 10, 16, 18, 25, 30, 39, 40]. As previously mentioned, this loss is partially due to the ability of the extraction process to break the bonds formed between the DNA and the swab material, as well as the ability of the swab to physically trap the DNA in its fibre matrix [3, 21, 22]. Additionally, the type of wetting agent used to moisten a swab can influence DNA extraction, with solutions such as SDS and water initiating cell lysis on the swab, thereby aiding the extraction process [7, 14, 41].

While previous studies have examined swab related variables influencing the recovery of biological material from surfaces, their limitations highlight the need for further investigation into touch DNA recovery. Much of the published research investigates swabs and wetting agents separately, with studies focusing on determining the most effective swab with only one wetting agent [2, 3, 6, 8, 19], and determining the most effective wetting agent using only one swab [12–15]. Therefore, simply selecting the top-performing swab type and wetting agent from the literature may not yield the most suitable combination in practice. Some investigations tested multiple swab types with multiple wetting agents and found that interactions between swabs and wetting agents can impact DNA recovery [7, 9, 11]. However, only one of these investigations examines touch DNA deposits, and in their study, only variation in wetting agent volume, not different wetting agent solutions, was assessed [11]. Currently, no investigation systematically evaluates the interactions between multiple swab types and multiple wetting agents for touch DNA recovery. Thus, information regarding the recovery of touch DNA remains limited. Furthermore, studies of touch DNA recovery (both single and multi-variable investigations) used volunteers to deposit touch DNA. However, volunteer-created touch deposits are, by their nature, inherently variable both between and within volunteers, and these studies do not account for the intra- and inter-person variability in the amount of DNA initially deposited [37, 42]. This unknown sample-to-sample variation in the amount of DNA initially deposited could skew the collected data, leading it to appear that the success of the variable under investigation (swab or wetting agent) influences DNA recovery, when in fact the influence comes from the DNA deposits. A synthetic touch DNA solution offers a possible alternative to body fluids or volunteer-produced deposits for studies requiring a known amount of starting material [43].

Although we have separately discussed variables that affect DNA recovery, it is essential to consider swab type, wetting agent, surface type, biological material, and the extraction process as a whole, as potential interactions among these variables influence overall DNA recovery differently than each variable alone [2, 3, 7, 9, 16]. Meaning, a swab and wetting agent combination needs to be specifically tailored for the recovery of a particular biological material, surface, and extraction protocol, where any deviations from this would require further investigation to identify differences in recovery efficiency.

Polypropylene is the second most widely produced synthetic plastic [44], and is extensively used in the manufacture of various items. As a result, polypropylene is frequently encountered as a surface of evidential significance, highlighting the need for an investigative focus on this surface type. In addition to its relevance at crime scenes, the collection of touch DNA from plastic also has implications for other forensically relevant research activities, specifically in investigations of DNA transfer and shedder testing, where polypropylene plastic tubes are commonly used as deposit surfaces [45–48]. Further, the DNA IQ™ extraction system is widely used in forensic laboratories internationally. Therefore, a touch DNA recovery method tailored for use on polypropylene surfaces, followed by swab extraction with the DNA IQ™ system, would be beneficial for both casework and research purposes. Therefore, this manuscript investigates variables to develop a standard workflow under these conditions.

This research aims to contribute to the development of a standardised touch DNA sampling method by proposing a swab and wetting agent combination workflow tailored for the recovery of touch DNA from polypropylene surfaces. To achieve this, we tested the recovery efficiency of five swabs (cotton, viscose, nylon, foam, and polyester) and three wetting agents (water, 2% SDS, and 100% isopropanol), in combination. We tested two relevant DNA deposit types: (1) a synthetic touch DNA deposit, containing a synthetic fingerprint solution, cellular DNA and cfDNA; and (2) naturally produced human-derived touch DNA deposits. Collection of these samples followed a systematic swab procedure, and we present this method to ensure consistency within the protocol.

## Materials and methods

### Experimental design

These experiments were conducted in two parts. Part one was conducted with a synthetic DNA deposition solution created to mimic touch DNA deposited through hand contact. Therefore, the starting amount of DNA in each deposit was known. Part two was conducted with natural touch DNA deposits, deposited by volunteers. Part two created a sample set in which the starting amount of DNA was unknown. A total of 150 DNA deposits (part one: *n* = 75; part two *n* = 75) were made (excluding controls) during this study. Before the commencement of experimental testing, a preliminary test of the methodology (including cell resuspension and the method and volume of wetting agent application) was conducted to ensure compatibility with the intended experimental protocol (Supplementary Material 1).

### Part one: synthetic touch DNA

#### DNA source

A synthetic DNA deposition solution of known concentration was created for use in part one. This solution was designed to mimic touch DNA deposits and consisted of cfDNA, cellular DNA, and a synthetic fingerprint solution [43].

The cfDNA component used in these deposits was a 10 ng/µL control DNA (Promega, Madison, USA), consisting of genomic DNA from a single male donor.

The cellular component of this mixture was provided by a single female donor. Thirty minutes prior to experimentation, the donor provided a 5 mL saliva sample collected in a clean 50 mL tube. The cells were separated from the saliva using a standard molecular biology methodology that does not damage the cells (Supplementary Material 1, Sect. 1.1). Briefly, saliva was thoroughly mixed, and an aliquot equivalent to the final master mix volume (sufficient to provide all deposit aliquots) was centrifuged at 10,000 g for 10 min. After centrifugation, the cell pellet was washed with 1,000 µL of 1X phosphate-buffered saline (PBS), pH 7.4 (Invitrogen, VIC, AU), and centrifuged again at 10,000 g for 10 min. After centrifugation, the cells were resuspended in 1X PBS, pH 7.4, by pipetting up and down until the mixture was homogeneous. This cell resuspension was then diluted 8-fold with the synthetic fingerprint solution.

The synthetic fingerprint solution, which acted as the suspension medium for the DNA components of this mixture, was an artificial eccrine-sebum emulsion (unstabilised) (BioChemazone, Alberta, CA). The emulsion was created to a 95% eccrine and 5% sebum mixture, which was balanced to a pH of 5.5 [49].

The synthetic touch DNA deposit solution was created by adding the cfDNA into the mixture of cellular DNA and the synthetic fingerprint solution. The concentration of the cfDNA in solution was 0.1 ng/µL, and the concentration of the cellular DNA was ~ 1.2 ng/µL. This solution was deposited on the target surface in 10 µL aliquots, yielding a total deposition of 1 ng cfDNA and ~ 12 ng cellular DNA. The amounts indicated for the cellular material have been calculated from control samples of the directly extracted master mix and, therefore, include DNA loss during the extraction process. These values are approximations, as cellular material is susceptible to clumping and small variations are characteristic of these kinds of solutions.

During sample deposition onto the polypropylene surface, 10 µL aliquots of the synthetic touch solution were also applied to glass microscope slides at four different timepoints. These were used to confirm the integrity of the cells in the solution used for part one of the experiment. One sample was deposited immediately after the DNA solution was made; two samples were deposited at different stages during the middle of sample deposition; and one sample was deposited at the end of the deposition. All samples were allowed to dry for at least 1 h prior to staining. A 1:500 solution of Diamond™ Nucleic Acid dye (DD) (Promega) was created from the 10,000X stock in a final concentration of 49% ethanol. This solution was then sprayed onto the four dried samples using a Voilamart air compressor (Voilamart™, Sydney, AU), held approximately 20 cm from the samples. These samples were allowed to dry in the dark for a minimum of 30 min. After the samples had dried, the deposits were visualised using a DinoLite microscope (EDGE AM4115T-GFBW, AnMo Electronics Corporation, New Taipei City, Taiwan) under 480 nm with an emission filter of 510 nm at 220X magnification.

#### Surface material, DNA deposition, and recovery

DNA was deposited on a flat, smooth polypropylene sheet (Plastics Australia, AU). This flat surface was used instead of a 50 mL tube, used in Part two, to prevent the solution from dripping down the tube’s rounded edges, which could have resulted in sample loss. Five 220 cm by 240 cm sheets were cut from the large primary sheet. Both sides of each sheet were washed with a 1% sodium hypochlorite solution, rinsed with deionised water, washed with 70% ethanol, rinsed again with deionised water, and patted dry using a Teri Wipe (Seton Australia, Sydney, AU) (Supplementary Material 2, Sect. 2.1). Once dry, a clean 3 by 5 grid consisting of an inner 3.5 cm by 3.5 cm square deposit guide was attached to the back of the polypropylene sheets (Supplementary Material 2, Sect. 2.2). These squares served as a guide for DNA deposition and indicated the area to be swabbed. A total of five deposit surfaces were created, one for each type of swab.

A total of 75 10 µL deposits of the synthetic touch DNA solution were pipetted as single drops (not spread) into the centre of the 3.5 cm by 3.5 cm squares. The surfaces were then allowed to dry for 24 h, protected in a fume hood (extractor off) before DNA collection. DNA was collected, comprising five replicates of each swab type (viscose, cotton, nylon, foam, and polyester) and wetting agent (water, IPA, and SDS) combination. This resulted in a total of 15 samples per swab type. Swabs were moistened with their designated volume of wetting agent (Materials and methods, Sect. 2.5), and a controlled single-swab method (Supplementary Material 2, Sect. 2.3) was used to collect DNA from the flat surface. The single-swab method was used instead of the double-swab technique, as the primary objective was to identify the combinations of swab type and wetting agent that facilitate increased DNA recovery. Since determining this optimum would benefit subsequent swabs, using a second swab was deemed unnecessary. Once swabbing was complete, the swabs were returned to their labelled containers for air drying and stored in paper envelopes (at room temperature) until DNA extraction (Materials and methods, Sect. 2.7).

Three types of positive controls were taken from the synthetic touch DNA deposit solution. The first, direct-to-buffer control was obtained by depositing 10 µL of the deposit solution (in triplicate) directly into a 1.5 mL tube and storing it at −20 °C until extraction. The second control, direct-to-dry swab, was prepared by depositing 10 µL of the deposit solution onto each type of dry swab (in triplicate). The third, direct-to-wet swab control involved moistening each swab type with the designated volume of each wetting agent, then depositing 10 µL of the synthetic touch DNA solution at the same location (in triplicate). All swab positive controls (dry and wet) were placed into their containers to dry and were stored in paper envelopes until DNA extraction. Negative controls of the wetting agents, synthetic fingerprint solution, 1X PBS and the surface used in part one of the study were also taken.

### Part two: volunteer-created touch DNA

#### DNA source

Touch DNA deposits used for part two of this experiment were donated by two female volunteers. All samples were collected with informed consent and human ethics approval by the Human Ethics Committee at Flinders University (Reference no. 4915). Volunteer one created 15 deposits per day on three separate days (45 deposits total), and volunteer two created 15 deposits per day on two separate days (30 deposits total). Volunteers were instructed to follow a strict handling procedure for DNA deposition, as detailed in Supplementary Material 2, Sect. 2.4.

#### Surface material and DNA deposition

In part two, 50 mL polypropylene conical centrifuge tubes (Tarsons, Parkway Process Solutions, VIC, AU) were used for volunteer-provided touch DNA deposits. Tubes were used instead of the flat surface from part one because sample dripping was not a concern. Additionally, holding a tube better represents items sampled for touch DNA in casework and other relevant activities, such as shedder testing, allowing DNA deposition from the whole hand during a natural holding action. Coloured swab guides were inserted into each tube, as shown in Supplementary Material 2, Fig. S2.5. These guides were used to divide the tubes into equal quarters, providing guidelines for the swabbing protocol, which is described in detail in Supplementary Material 2, Sect. 2.5. Tubes were then washed with 1% sodium hypochlorite solution, rinsed with deionised water, washed with 70% ethanol and rinsed again with deionised water. The tubes were then irradiated with UV light (BLX Bio-Link Crosslinker, Vilber) for 30 min and stored in a clean plastic bag until use.

For the deposition of the natural touch DNA samples, volunteers were asked to go about their normal day (without wearing gloves, using the bathroom, washing their hands, or eating) for one hour before sample deposition. Immediately before deposition, volunteers were asked to rub the palmar surface of their hands together in a back-and-forth motion three times. This was done to equalise the DNA on the surfaces of the hands. They were then asked to contact one of two (for contact with right and left hands) 50 mL conical centrifuge tubes suspended in air using a retort stand, following the prescribed procedure, described in detail in Supplementary Material 2, Sect. 2.4. Two deposits were completed at each deposition time, one for each hand. After the deposition, volunteers were instructed to return to their daily routine, with the conditions described above being met for a period of one hour prior to the next deposition. Omitting a hand-washing step between deposits allowed for maximal accumulation of DNA on the hands before deposition. Sufficient DNA accumulation is important, as deposits with no detectable DNA are not conducive to a recovery study. After both depositions, the tubes were removed from the retort stand and stored (upright on their lids), protected in a fume hood (extractor off), to dry for 24 h before sample collection. New, clean tubes were then secured in place on the retort stand, ready for the next deposit 1 h later. Eight deposition events were conducted in a day (each retort stand and set of clamps was assigned to one volunteer, and each apparatus was cleaned before first use and after each day), resulting in a total of 15 deposits per volunteer (only one deposit was made in the 8th deposit event). In total, 75 touch DNA samples were collected from the volunteers over three days.

#### DNA recovery

Seventy-five volunteer-provided touch DNA deposits were created, resulting in 150 collected samples, 75 test swabs and 75 control swabs. Five replicates of each test swab (viscose, cotton, nylon, foam and polyester) and wetting agent (water, IPA, SDS) combination were collected from one half of a 50 mL tube, as defined using the coloured swab guide shown in Supplementary Material 2, Sect. 2.5. These samples will further be referred to as the test swab samples. The remaining half of the tube was swabbed with a viscose swab (selected because it is the swab type currently used by forensic services in our jurisdiction for the collection of touch DNA samples) and the corresponding wetting agent; these samples will be referred to as the control swab samples (Supplementary Material 2, Sect. 2.5). Swabs were moistened with the designated volume of wetting agent (Materials and methods, Sect. 2.5), and a controlled single-swab method (outlined in detail in Supplementary Material 2, Sect. 2.5) was used to recover DNA from the surface of the 50 mL tubes. Control swabs were used to adjust for sample-to-sample variation in the starting amount and distribution of touch DNA between individuals and replicates; this calculation is described in the Results, Sect. 3.2.

### Swabs

**Fig. 1:**
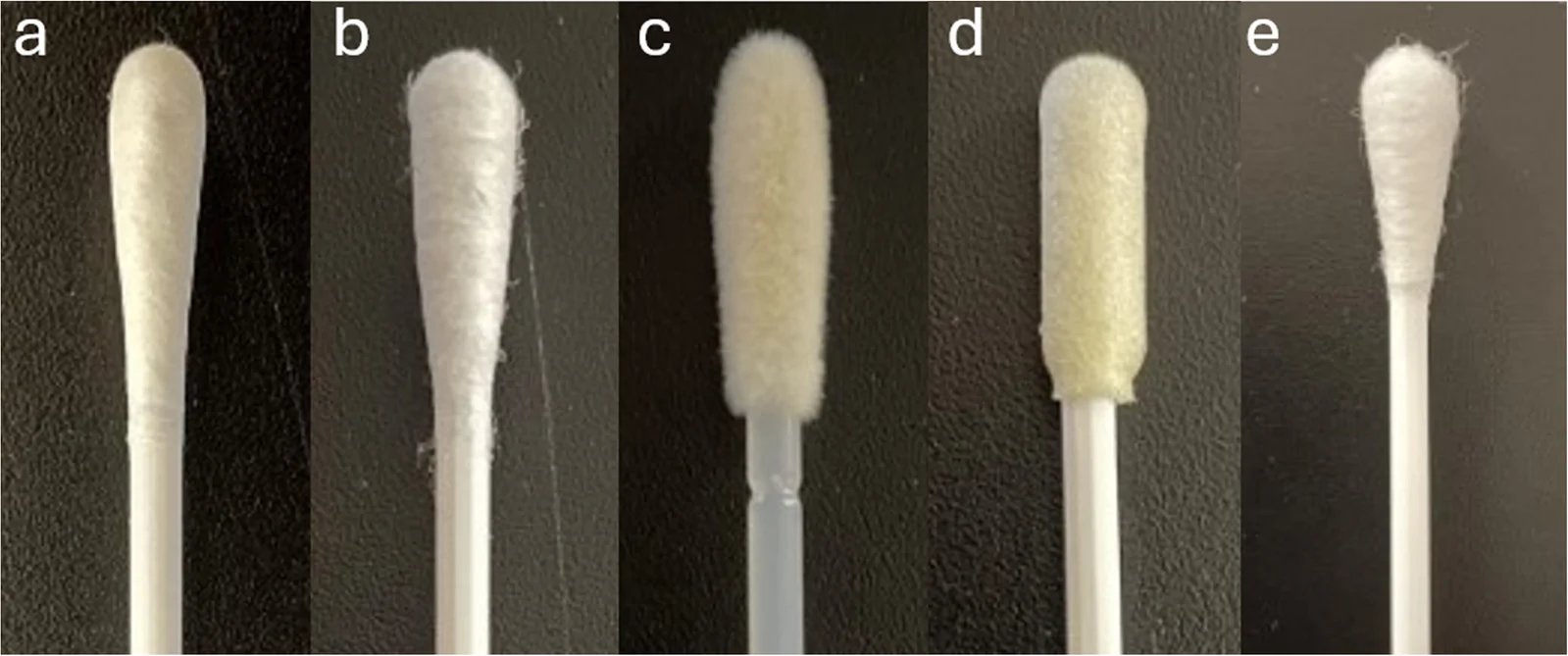
Close-up of swab heads. **a**) Sarstedt, viscose; **b**) Puritan, cotton; **c**) Copan, nylon; **d**) Puritan, foam; and **e**) MWE, polyester

Five swabs of different head materials were used in this experiment, and details of these swabs can be found in Table 1. Images of the swabs and their packaging can be found in Supplementary Material 2, Sect. 2.6 and close-up images of the swab heads can be seen in Fig. 1. Swabs without ventilation built into the container (nylon, foam and polyester) were vented manually using a scalpel to make a small incision at the end of the container. The swabs chosen for testing in this experiment were selected based on two criteria. First, swabs were selected based on the success of the swabbing material in collecting DNA, as outlined in the literature [2, 3, 6–8, 11]. One swab of each material was selected, and, where multiple swabs of the same material were available, those commonly used in the literature or casework were chosen. Second, this selection was also informed by anecdotal reports of forensic laboratories worldwide using these swabs in casework.

**Table 1:** Specifications of swabs used for DNA recovery. Information includes purchasing details, swab dimensions (length (L) and diameter (D), packaging descriptions, and anti-contamination features

				Head					Shaft			Anti-Contamination		
Product specification	Abbreviation	Brand	Reference	Material	Arrangement of fibres	Size (mm)	Sterility	Standard	Composition	Size (mm)	Overall length (mm)	Packaging	Storage	Drying system
Forensic swab, L, round, in the tube with ventilation membrane, ISO 18,385, 85 mm, viscose	Viscose	Sarstedt	80.630	Viscose	Coiled	L 18.5 D 3.0	ETO treated- DNA Free	ISO 18,385	Polystyrene	L 59.0 D 2.0	164.5	Box of swabs, all in individual containers	Container	Membrane
Fab-Swab 5” Sterile DNA Controlled Standard Cotton Swab & Transport Tube- Fab-Swab (Filtered air breathable) DNA controlled regular cotton tipped applicator with plastic handle	Cotton	Puritan Medical	25–805 1PC TT FABUSA	High quality USP Cotton	Coiled	L 17.5 D 5.2	DNA free	ISO 9001:2008 ISO 13485:200З	Polystyrene	L 109.0 D 2.5	1370	Box of swabs, all in individual containers, individually wrapped in plastic	Container	Filter
4N6FLOQSwabs™, regular tip, tube	Nylon	Copan	4,479,433	Nylon	Coated with perpendicular fibres	L 15.0 D 4.0	DNA free	ISO 18385:2016	Plastic	L 121.0 D 3.0	168.0	Box of swabs, all in individual containers	Container	No built-in drying system, manual aeration needed
Puritan 25–1506 1PF BT BL Cell Collection Foam Swab with Transport Tube	Foam	Puritan Medical	P25-1506-1PF-BT	Medical grade polyurethane foam	100 pores per inch	L 16.0 D 4.0	Sterile	ISO 9001:2008 ISO 13485:200З	Polystyrene	L 123.0 D 2.5	171.5	Box of swabs, all in individual containers	Container	No built-in drying system, manual aeration needed
Medical Wire MW102D Swab Plastic Stick	Polyester	Medical Wire (MWE)	B22721-ETO	Polyester (Dacron)	Coiled	L 17.5 D 5.0	ETO treated- DNA Free	ISO 18385:2016 ISO 17020:2012	Plastic	L 110.0 D 2.0	160.0	Box of swabs, all in individual containers	Container	No built-in drying system, manual aeration needed

### Wetting agents

Three wetting agents were used in this experiment, and details are listed in Table 2. The wetting agents chosen for testing in this experiment were selected based on two criteria. First, wetting agents were selected based on the success of these solutions in recovering DNA, as indicated by previously published work [7, 9, 12–14], and second, based on published information indicating that forensic laboratories may use some of these agents in casework [5, 9, 34, 50].

**Table 2:** Wetting agents used to moisten swabs

Wetting agent	Abbreviation	Concentration used	Supplier	Reference	Concentration purchased
Water for Injections BP	Water	n/a	Medisa	158,596	n/a
Isopropanol (2-Propanol)	IPA	100%	RCI Labscan	AR1162	100%
Sodium dodecyl sulphate	SDS	2% *	Thermo Fisher Scientific	AM9820	20%

* SDS was purchased at 20% and was diluted with deionised water to create the 2% wetting agent solution

### Swab moistening

In both parts of these experiments, swab heads were moistened before DNA collection from the target surface. Preliminary testing confirmed that the most controlled mode of wetting agent deposition was via a micropipette (Supplementary Material 1, Sect. 1.2), and the volume of wetting agent required for each swab type (Supplementary Material 1, Sect. 1.3). The wetting agent volumes used with each swab type for DNA collection in parts one and two of this experiment are shown in Table 3. During sample collection, general observations on the ease of use of each swab type and wetting agent, cost, availability, and moisture absorption were recorded; details of these observations are provided in Supplementary Material 3, Sect. 3.1.

**Table 3:** Swab type and corresponding volume of wetting agent required for moistening

Swab brand	Head material	Wetting agent volume
Sarstedt	Viscose	50 µL
Puritan	Cotton	50 µL
Copan	Nylon	10 µL
Puritan	Foam	10 µL
MWE	Polyester	50 µL

### DNA analysis

DNA was extracted from the swabs using the DNA IQ™ (Promega, USA) system and a manual extraction protocol, following the manufacturer’s guidelines, with slight modifications. At the lysis step, 500 µL of lysis buffer was used to fully cover each swab head, and lysis was performed while shaking at 650 RPM. Due to the increased lysis buffer volume, the magnetic resin volume was increased to 10 µL, and the samples were incubated for 40 min at 37 °C. Modifications to the wash steps reduced the method from three washes to two with 1X wash buffer: the first with 300 µL and the second with 100 µL. Elution was completed to a volume of 60 µL and stored at 4 °C until quantification. Samples were quantified using Quantifiler™ Trio kit (Applied Biosystems, USA) on an ABI- PRISM™ 7500 (Life Technologies, USA) according to the manufacturer’s recommendations.

### Data analysis

The amount of DNA recovered from the surface with each swab and wetting agent combination was calculated by multiplying the DNA quantity determined during quantification by 60 µL (volume of eluent) (Supplementary Material 4). In part one, the DNA extraction efficiency was calculated for each swab. To determine extraction efficiency from dry swabs, the average total DNA quantity (ng) obtained from three direct-to-dry swab controls was divided by the average total DNA quantity (ng) obtained from three direct-to-buffer samples, then multiplied by 100, and reported as a percentage. These calculations were repeated for the cellular fraction of the DNA mixture (total DNA - Y DNA). For the cfDNA fraction of the mixture (Y DNA), the total DNA input was known to be 1 ng; therefore, the DNA recovery amounts (ng) from the direct-to-dry swabs were divided by one (instead of being divided by the direct-to-buffer value), then multiplied by 100 and reported as a percentage. All calculations for the direct-to-wet swab controls were carried out in the same manner; however, since only one sample was analysed, the wet swab control value was not averaged.

Quantification values were log10 transformed, and samples with 0 ng of recovered DNA were assigned a value of 0.00005 ng to allow transformation of these data points to the log10 scale. A multiple linear regression was performed (IBM SPSS Statistics, version 29.0.1.0) to examine whether swab type, wetting agent, and their subsequent interactions impacted DNA recovery. An ANOVA with a Tukey post hoc test (IBM SPSS Statistics, version 29.0.1.0) was used to determine whether differences among the swab and wetting agent combinations were statistically significant.

Five replicates per swab and wetting agent limits the ability to detect effects. Small sample sizes become problematic when effects are subtle, variance in the data is high, or models are over-parameterised. If effects are large and consistent, meaningful inference can still be possible. A number of steps were taken to mitigate any sample size issue:

- Analysis was carried out using regressions on the full datasets of 75 samples for each part. This means that conclusions were not drawn from comparison between 15 groups of five, but rather the regression was able to borrow learning from across the entire dataset for all inferences.
- A combination of Bayesian Information Criteria (which works to counter spurious conclusions from smaller datasets), variable significance, and domain knowledge were used to choose the appropriate model and minimise overfitting of any model or over-reliance on traditional (non)significance alone (which again, can be volatile for low sample numbers and highly variable data).
- Effect sizes were calculated for variables, in order to ensure that when inferences are being drawn from the regression results, they do not rely on small effect sizes (which again must be treated with particular caution in low sample-size datasets).

## Results

### Part one: synthetic touch DNA deposits

#### Part one: Cell integrity

The results of the cell integrity check of the DNA deposition solution used to create the part one samples indicated that the buccal cells were intact; the results are available in Supplementary Material 3, Sect. 3.2 and 3.3.

#### Extraction efficiency from control swabs

The extraction efficiency (elution of the sample from the swab) was determined in two ways using the positive controls collected (Materials and methods, Sect. 2.2.2) in part one of this experiment. First, the dry control swabs were used to identify which head fibres have the most efficient DNA release capabilities during extraction with the DNA IQ™ system. Second, wet control swabs were used to determine whether the addition of a wetting agent would affect the extraction efficiency of a swab.

**Fig. 2:**
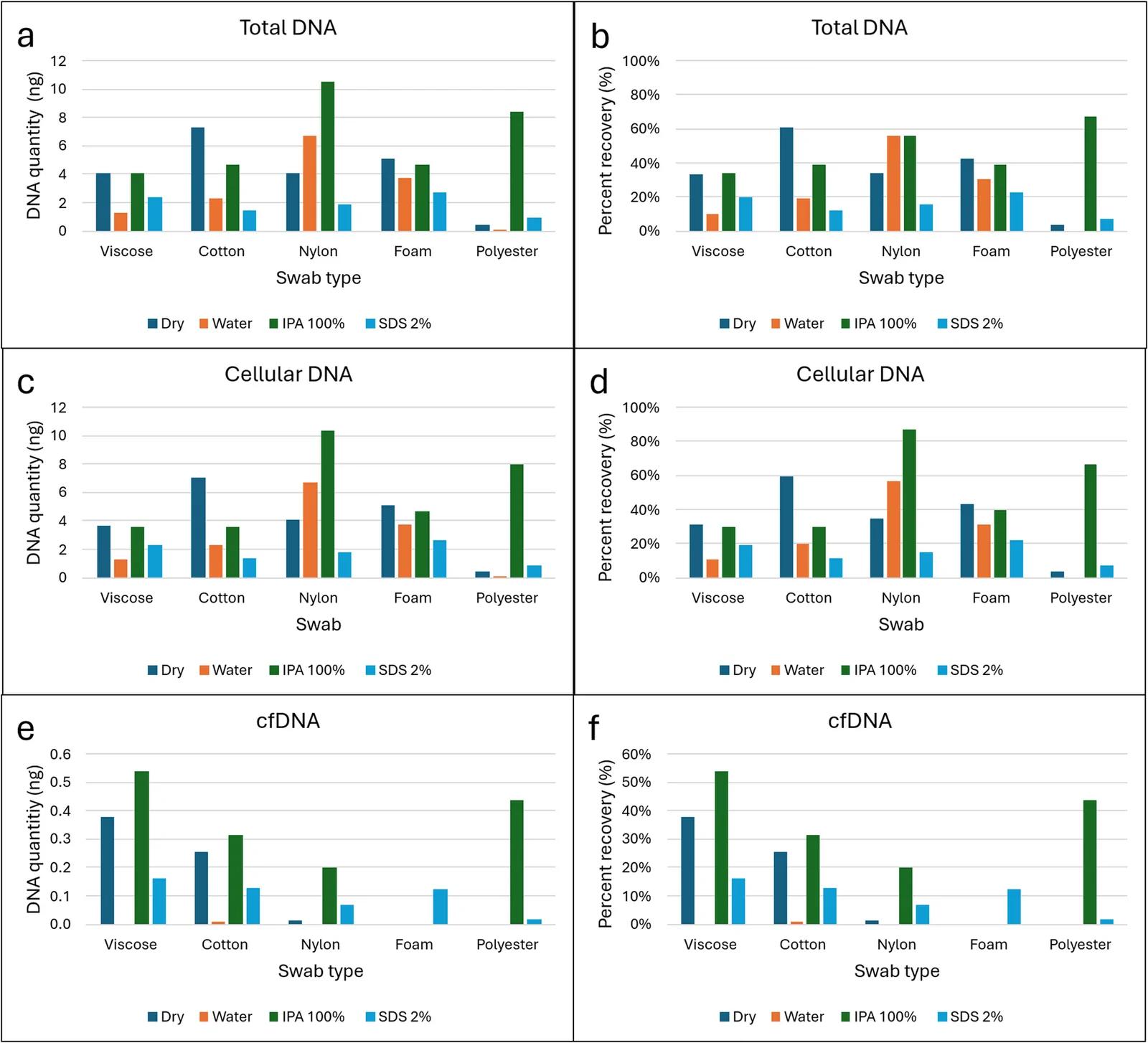
Extraction efficiency from all DNA types, swab type and wetting agent. Figures **a**, **c**, and **e** present the extraction efficiency data in total amount (ng) for total, cellular, and cfDNA, respectively. Figures **b**, **d** and **f** present the extraction efficiency as percent recovery for total, cellular, and cfDNA, respectively. Values for dry swabs are the average of triplicate samples, whereas the values for the wetting agent are obtained from a single swab

The DNA recovered from all positive controls used in this study is shown in Fig. 2 (raw data shown in Supplementary Material 4, Table 1). Considering extraction efficiency from dry swabs, with the exception of polyester, which showed DNA recovery below 5%, all swab types exhibited recovery between 31% and 61% for total and cellular DNA (Fig. 2b and d). The recovery efficiency of cfDNA was lower than that of cellular for nylon, foam, and polyester swabs, yielding less than 1.5% recovery (Fig. 2f). However, cotton and viscose had higher extraction efficiencies, yielding 26% and 38% recovery, respectively (Fig. 2f).

**Fig. 3:**
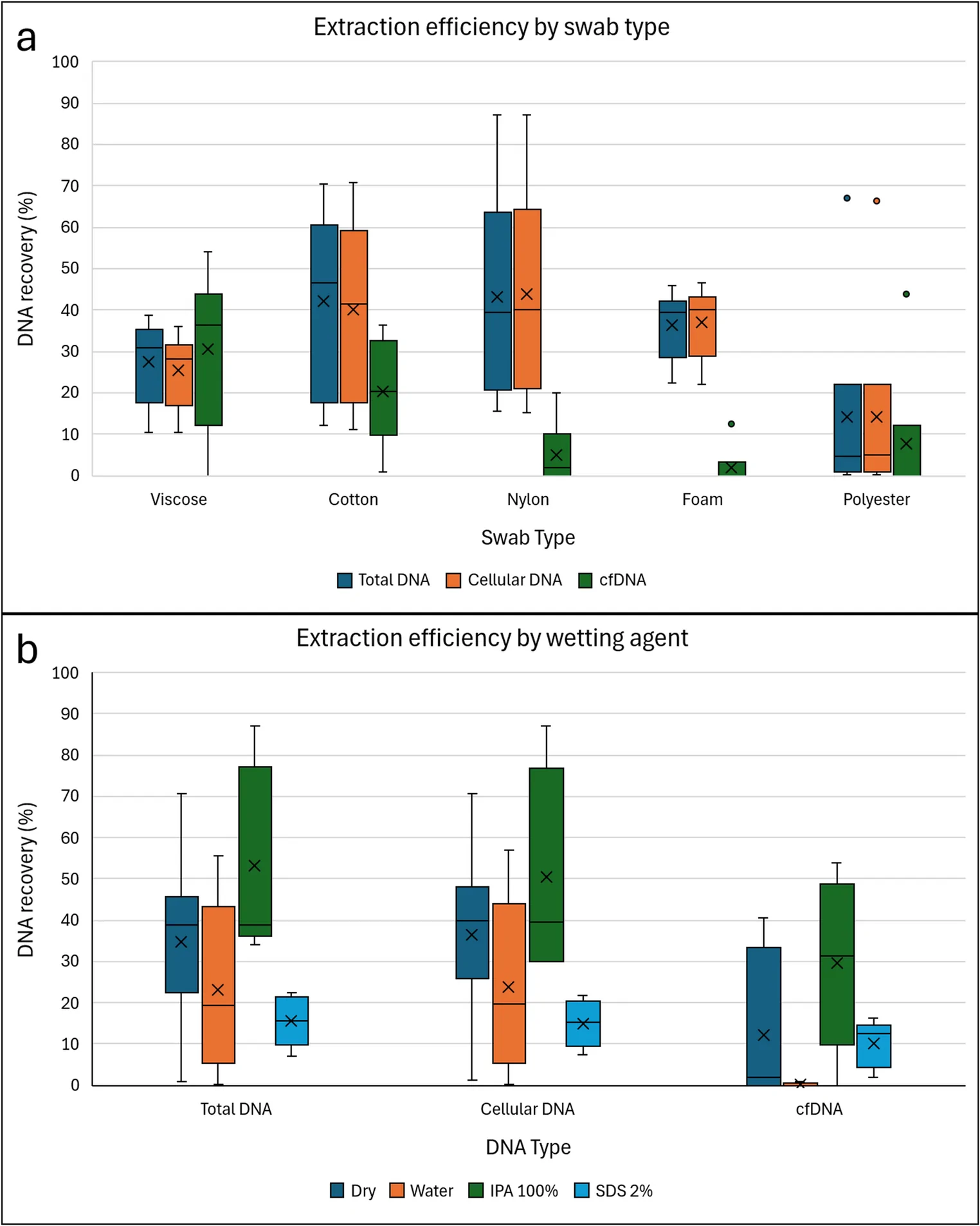
Extraction efficiency by swab type and wetting agent; **a**) extraction efficiency of each DNA type when considering only the swab type (*n* = 6), and **b**) extraction efficiency of each DNA type when considering only the wetting agent (dry; *n* = 15, water; *n* = 5, IPA 100%; *n* = 5, SDS 2%; *n* = 5). Where x indicates the mean

When testing the extraction efficiency of swabs moistened with the most commonly used wetting agent, water, it was found that the polyester swab yielded no recovery for any of the DNA types (Fig. 2). All other swabs yielded extraction efficiencies ranging from 10% to 57% for both total and cellular DNA (Fig. 2b and d). The addition of water dramatically decreases cfDNA recovery from viscose and cotton when compared to dry swabs, with no water-moistened swabs providing cfDNA recovery percentages over 1% (Fig. 2f). When the swab type is not considered, the extraction efficiency of dry swabs across all DNA types was greater than that of swabs moistened with water (Fig. 3b).

In general, the addition of SDS produced low extraction efficiencies (7%−22%) for both total and cellular DNA (Fig. 2b and d). Considering cfDNA, the addition of SDS increased DNA yield over all water-moistened swabs and over dry swabs for nylon, foam, and polyester; however, the overall DNA yield remained low (2%−16%) (Fig. 2f). When the swab type is not considered, the extraction efficiency of SDS moistened swabs was higher than observed with dry or water moistened swabs for cfDNA. However, for total and cellular DNA, SDS moistened swabs yielded comparable results to water moistened swabs, but less than dry or IPA moistened swabs (Fig. 3b).

On average, the use of IPA as a wetting agent increases extraction efficiency compared to dry or water-moistened swabs (Fig. 3b), reaching 30%−87% (Fig. 2b and d). All IPA moistened swabs exhibited a marked increase (over dry or water moistened swabs) in cfDNA yield, with extraction efficiencies ranging from 20% to 54% (Fig. 2f). The only exception to this was the foam swabs, in which the extraction efficiencies remained 0%. When the swab type was not considered, the extraction efficiency of IPA-moistened swabs was higher than that observed from dry, water-moistened or SDS-moistened swabs for each DNA type (Fig. 2b).

Generally, the extraction efficiency of swabs in releasing total and cellular DNA was within the expected range; however, it was influenced by the type of wetting agent used. When considering the extraction efficiency of different DNA types from swabs, without accounting for the influence of wetting agents, cotton, nylon and foam, all exhibit similar recoveries for total and cellular DNA (Fig. 3a). Viscose swabs tend to show slightly lower recoveries of total and cellular DNA; however, they exhibit the highest extraction efficiency for cfDNA and consistently exhibit the least variation (25–30%) in extraction efficiency across all DNA types (Fig. 3a). When compared to the recovery of cellular DNA, nylon and foam swabs exhibited reduced extraction efficiency for cfDNA. Cotton and polyester also showed a decrease in cfDNA yield compared with cellular DNA; however, the recovery difference between the DNA types was less pronounced than that observed with nylon and foam (Fig. 3a). For example, for nylon swabs, the recovery efficiency difference between cellular and cfDNA was 39% compared to cotton, where a smaller difference of 20% was observed (Fig. 3). Polyester swabs yielded the lowest overall extraction efficiencies among the different swab types. Furthermore, when considering the extraction efficiency of different DNA types with different wetting agents, without accounting for the influence of swab type, 100% isopropanol tended to yield the highest DNA recovery rates (Fig. 3b).

#### Swab and wetting agent interactions

**Fig. 4:**
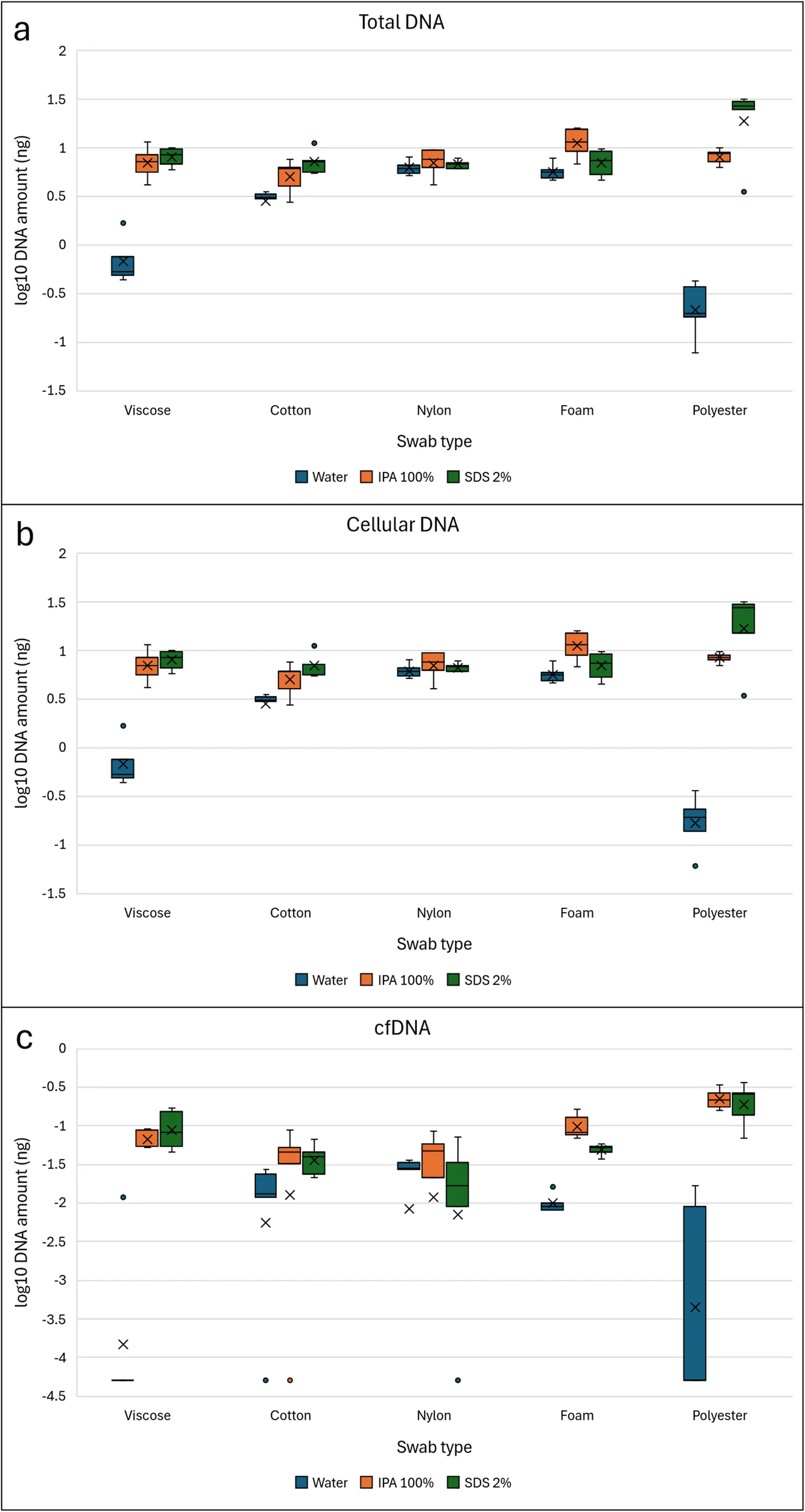
DNA recovery (shown on a log10 scale) for samples collected using each combination of swab type and wetting (*n* = 5) in part one. Where **a**) represents total DNA, **b**) cellular DNA, and c) cfDNA. Where x indicates the mean

Several different regression models were tested (considering only main effects and no interactions) prior to the model described below. Final model choice was based on Bayesian Information Criteria (BIC). The final model for total and cellular DNA was an interaction model, performed using multiple linear regression (raw data (Supplementary Material 4, Table 2) shown graphically in Fig. 4). The fitted regression model was:

$$\:Y={\beta\:}_0+{\beta\:}_{1,\:i\:}S_i+{\beta\:}_{2,\:j}W_j+{\beta\:}_{3,I,j}\:\left(S_iW_j\right)$$

where Y is the total DNA amount obtained (in log10(ng)), S_i_ is an indicator term for swab type, W_j_ is an indicator term for wetting agent, and β are the coefficients. The results of the linear regression for total DNA showed that swab type (*p* < 0.001, effect size d = 1.86), wetting agent (*p* < 0.001, effect size d = 3.90), and their interaction (*p* < 0.001, effect size d = 3.82) were all significant predictors in the model. The results of the linear regression for cellular DNA showed that swab type (*p* < 0.001, effect size d = 1.84), wetting agent (*p* < 0.001, effect size d = 3.80) and their interaction (*p* < 0.001, effect size d = 3.74) were all significant predictors in the model. The final model, with the lowest BIC, for cfDNA was a ‘main effects only’ model. However, given multiple significant interaction terms and the significance of interaction in the other models, we have chosen, for consistency, to continue using the interaction model for this data. The results of the linear regression for cfDNA showed that swab type (*p* = 0.225, effect size d = 0.62), wetting agent (*p* < 0.001, effect size d = 1.66) and their interaction (*p* = 0.002, effect size d = 1.38) were all significant predictors in the model. In the total and cellular DNA models, cotton, nylon, and foam swabs recovered significantly more DNA than viscose. Additionally, IPA and SDS were found to recover significantly more than water. However, the inclusion of interaction terms showed that the effect of swab type varied by wetting agent, and vice versa, with two combinations associated with significantly higher yield (polyester and IPA; *p* = 0.001 and polyester and SDS; *p* < 0.001). For cfDNA, all combinations were associated with significantly lower yield, except for polyester and IPA, which were not significantly higher (*p* = 0.957), and polyester and SDS, which were not significantly lower (*p* = 0.841). The regression model was used to generate a predicted DNA recovery for each of the combinations of swab and wetting agent. The results of this analysis are presented in order from highest (1st) to lowest (15th) DNA recovery in Table 4. We note that the difference in predicted DNA amounts between adjacent (or even further separated) entries in the ranked list of Table 4 can be small and therefore, given the sample size of our study, should only be considered at a general trend level rather than an authoritative, unchanging ranked list.

Overall, these results found that the swab and wetting agent interaction terms were significantly important in predicting total and cellular DNA recovery, but less important in predicting the recovery of cfDNA. Overall, across all data, polyester swabs moistened with SDS consistently ranked among the top-preforming combinations. However, the difference in DNA yield was not always statistically significant, and significance appeared to depend on the type of DNA.

**Table 4:** Results of the multiple linear regression interaction model. Swab and wetting agent pairs are ranked 1–15, with 1 st indicating the combinations where the highest quantity of DNA was recovered, and 15th the combinations where the lowest quantity of DNA was recovered

Rank	Part One			Part Two
	Total DNA	cfDNA	Cellular DNA	Total DNA
1st	Polyester and SDS	Polyester and IPA	Polyester and SDS	Cotton and SDS
2nd	Foam and IPA	Polyester and SDS	Foam and IPA	Polyester and SDS
3rd	Polyester and IPA	Foam and IPA	Viscose and SDS	Cotton and IPA
4th	Viscose and SDS	Viscose and SDS	Polyester and IPA	Viscose and IPA
5th	Cotton and SDS	Viscose and IPA	Cotton and SDS	Nylon and water
6th	Nylon and IPA	Cotton and SDS	Nylon and IPA	Viscose and SDS
7th	Viscose and IPA	Cotton and IPA	Viscose and IPA	Viscose and water
8th	Foam and SDS	Nylon and IPA	Nylon and SDS	Foam and SDS
9th	Nylon and SDS	Foam and water	Nylon and water	Polyester and water
10th	Nylon and water	Nylon and water	Foam and SDS	Nylon and SDS
11th	Foam and water	Foam and SDS	Foam and water	Polyester and IPA
12th	Cotton and IPA	Nylon and SDS	Cotton and IPA	Cotton and water
13th	Cotton and water	Cotton and water	Cotton and water	Nylon and IPA
14th	Viscose and water	Polyester and water	Viscose and water	Foam and IPA
15th	Polyester and water	Viscose and water	Polyester and water	Foam and water

### Part two: volunteer-created natural touch DNA deposits

At the conclusion of part two, there were 150 swabs taken, 75 each of controls and test samples. The control samples were always viscose swabs but varied in the wetting agent used. We wished to account for sample-to-sample variation using the control swabs so that (a) individual replicate results within a wetting agent could be compared and (b) results between wetting agents could be compared. To account for the former (sample-to-sample) variation the DNA amount (ng) from the test swab of each sample was divided by the DNA amount (ng) from the control swab. To account for the latter (difference in wetting agent used on control swabs) the ratio of DNA amounts was then multiplied by the average DNA amount (ng) for the corresponding wetting agent controls (e.g., if considering a water test sample, the ratios were multiplied by the average DNA amount of all viscose control swabs moistened with water, *n* = 25).The resulting scaled ratio quantity (which can again be considered adjusted ng of DNA) was then log10 transformed. Four outliers (one replicate of viscose and water, one of viscose and IPA, and two replicates of nylon and water) were identified and removed for downstream analysis (Supplementary Material 4, Table 3). Outliers were identified as values falling outside 1.5 times the interquartile range (IQR) of the dataset. Outliers arose from large, corrected values caused by control swabs recovering substantially less DNA than test swabs, thereby violating the assumption of our sample-to-sample correction that DNA is evenly distributed across the surface. In instances where DNA is unevenly distributed, choosing a swab and wetting agent combination could be skewed without outlier removal.

#### Swab and wetting agent interactions

**Fig. 5:**
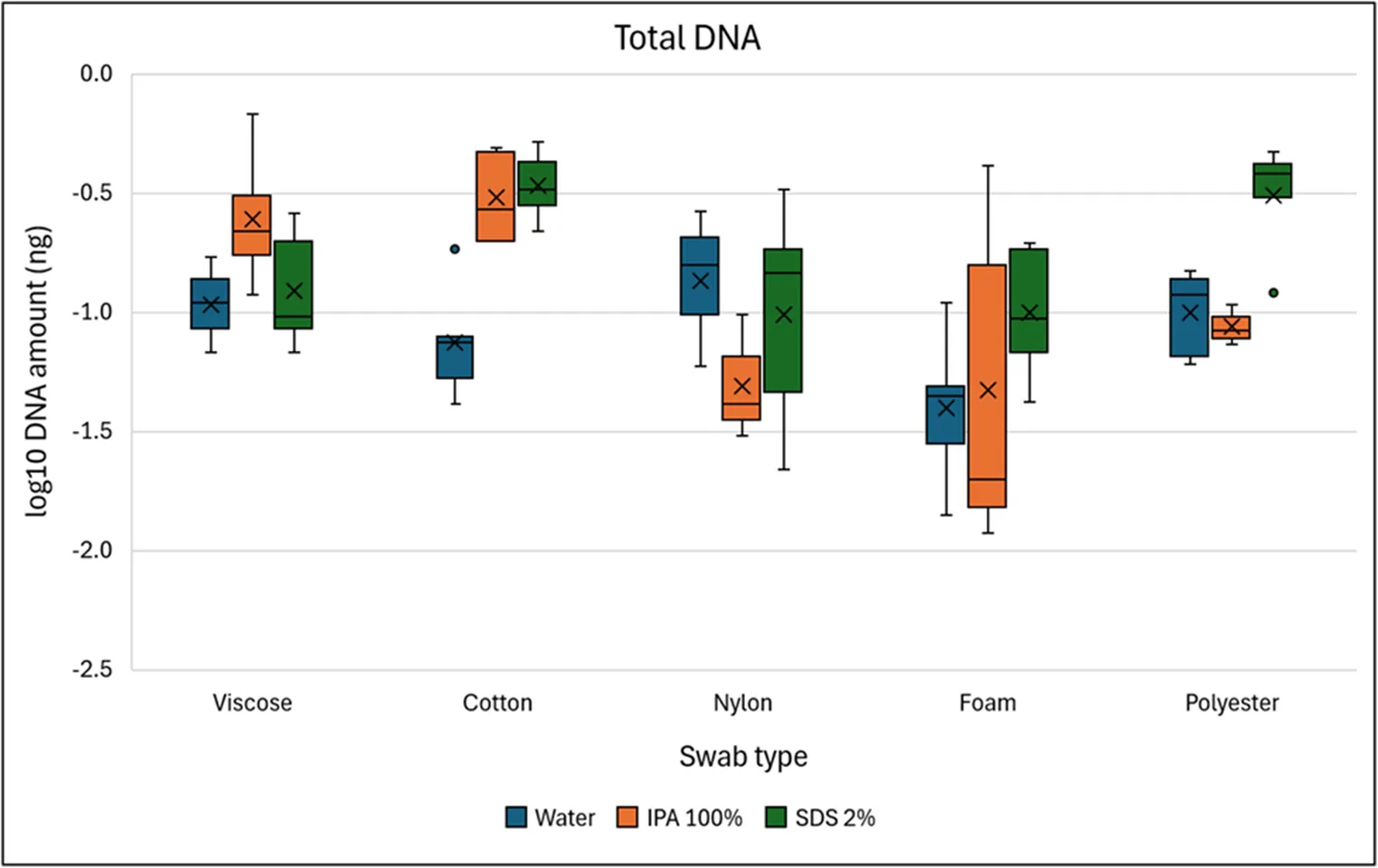
DNA recovery (shown on a log10 scale) for samples collected using each combination of swab type and wetting in part two (*n* = 5). Where x indicates the mean

Several different regression models were tested (considering only main effects and no interactions) prior to the model described below. The selected model did not have the lowest BIC; however, it did include significant interaction terms and, given the importance of these terms in the other models, it is reasonable to assume they may be important to include. Therefore, we have included them again here. This final model was an interaction model, performed using multiple linear regression (raw data (Supplementary Material 4, Table 3) shown graphically in Fig. 5). The fitted regression model was

$$\:Y={\beta\:}_{0}+{\beta\:}_{1,\:i\:}{S}_{i}+{\beta\:}_{2,\:j}{W}_{j}+{\beta\:}_{3,I,j}\:\left({S}_{i}{W}_{j}\right)$$

where Y is the total DNA amount obtained (in log10(ng)), S_i_ is an indicator term for swab type, W_j_ is an indicator term for wetting agent, and β are the coefficients. The results of the linear regression showed that swab type (*p* < 0.001, effect size d = 1.42), wetting agent (*p* = 0.003, effect size d = 0.96), and their interaction (*p* = 0.011, effect size d = 1.26) were all significant predictors in the model. The results of this analysis are presented in order from highest (1st) to lowest (15th) DNA recovery in Table 4. In this model, the inclusion of interaction terms showed that the effect of swab type varied by wetting agent and vice versa, with one combination associated with significantly higher yield (cotton and SDS, *p* = 0.014) and another with significantly lower yield (nylon and IPA, *p* = 0.043). Note that in this model, we are not looking directly at DNA amounts but rather amounts that have been adjusted to take into account sample-to-sample effects during deposition. This can be thought of as a proxy for DNA amounts (in the absence of sample-to-sample effects), and we simply refer to DNA amounts when we speak of the modelled factor.

**Fig. 6:**
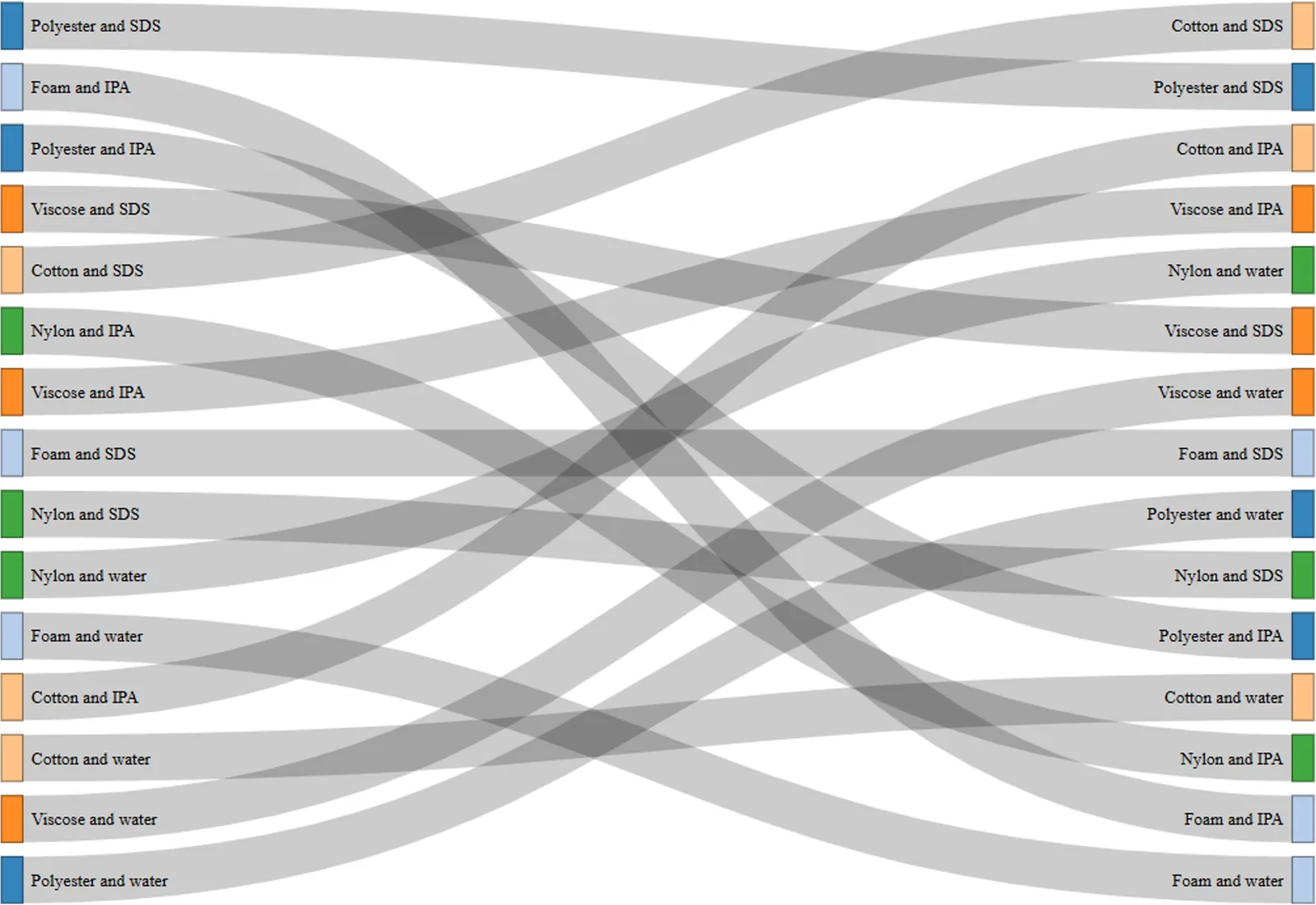
Sankey diagram showing the changes in rank for each swab and wetting agent combination between synthetic and natural deposits. The left column represents the ranking of combinations from Table 4, part one (total DNA, synthetic deposits), and the right column represents the ranking from part two (total DNA, natural deposits). Grey connecting bands illustrate the change in rank for each combination between the two sample types

Overall, these results showed that the swab-wetting agent interaction terms were significantly important in predicting DNA recovery. The data suggest that cotton swabs moistened with SDS rank among the best-performing combinations for DNA recovery. However, the difference in DNA yield was not always statistically significant. Differences in the rankings of swab and wetting agent combinations for parts one and two (total DNA) are shown in Fig. 6.

## Discussion

### Extraction efficiency

When swabs were dry, cotton swabs were found to have the highest extraction efficiency for total DNA, followed by foam, nylon, viscose, and polyester. In the literature, the extraction efficiency of cotton swabs is somewhat unclear; however, it is generally accepted that they effectively collect DNA but do not effectively release it during the extraction process. Some studies provide evidence in support of this notion [9, 10, 21], while others contradict it, finding that cotton swabs exhibit an equal or superior extraction efficiency to other swab types [3, 6, 8, 16]. On the other hand, foam and nylon floq swabs are generally considered to have superior DNA release capabilities; however, like with cotton swabs, the literature both supports [3, 6, 10, 16] and contradicts [8, 51] this. Our results show that polyester exhibited the lowest extraction efficiency among the tested swabs, which was consistent with the findings of Verdon et al. [3], who reported poor extraction efficiency for blood and saliva with the same MWE polyester swab. In their study, the polyester swabs used to assess extraction efficiency were moistened with water before the biological material was directly deposited onto the swabs [3]. Our results show that adding water to the polyester swab prior to DNA deposition did not increase extraction efficiency for any DNA type compared with dry swabs, remaining consistent with Verdon et al. [3]. However, it is important to note that previous studies have used body fluids to assess the extraction efficiency of swabs, not touch DNA samples, limiting the ability to extrapolate this data to infer extraction efficiencies for touch DNA samples. Comment et al. [8] noted that conducting an investigation of extraction efficiency with body fluids will yield results unlikely to be comparable to touch DNA samples; however, they highlight that standardising touch DNA samples for extraction efficiency testing is extremely difficult. This knowledge gap is further highlighted by a recent review by the National Institute of Justice [52], which emphasised that few studies have investigated swab materials for the extraction efficiency from cfDNA samples, and none have explored touch DNA. Assessing extraction efficiency with touch DNA samples is challenging due to the nature of the volunteer deposit; however, it has been previously accomplished using a synthetic touch DNA solution that was deposited onto a dry cotton swab yielded reasonable extraction efficiencies for both cellular DNA (55%) and cfDNA (30%) [43]. Our results are consistent with this previous finding, as dry cotton swabs yielded extraction efficiencies of 60% and 25% for cellular and cfDNA, respectively. By using a synthetic touch DNA solution, we have obtained extraction efficiency results for the tested swabs and wetting agents, with samples that more closely mimic natural touch DNA deposits than is possible with body fluids or biological materials deposited in a buffer (e.g. 1X PBS). We encourage the continued use of similar synthetic deposits for testing DNA behaviour when standardised protocols and control conditions are required. However, comparison of the recovery results from parts 1 and 2 of this study reveals differences between synthetic and natural touch samples, suggesting the presence of additional factors in touch deposits that are not accounted for in synthetic samples (discussed further in Discussion, Sect. 4.2). These findings highlight the need for a deeper understanding of the chemical and biological composition of natural touch deposits, which could inform refinements to synthetic solutions to more accurately represent natural deposits. For instance, changes that would make deposit volumes more comparable with the amount of liquid left behind during natural hand contact may help align synthetic touch with natural touch deposits.

Many previous studies measured extraction efficiency on a dry swab or a swab moistened with water; however, as we have shown, wetting agents can impact the DNA recovery efficiency, which encompasses extraction efficiency. Thus, it is important to understand the extent to which wetting agents affect the extraction step. Therefore, when investigating the impact of wetting agents, it is important to take control samples that represent moistened swabs. A study by Krosch et al. [9] collected positive controls from all wetting agent and swab combinations tested; however, they did not assess the extraction efficiency from these samples, nor did they provide the starting concentration of the body fluid dilutions, so the extraction efficiency could not be calculated. This type of information would be beneficial to the wider scientific community, as our current understanding of how different wetting agents affect extraction efficiency is limited. Our results suggest that including wetting agents as positive controls used to determine extraction efficiency will affect DNA yield (Fig. 3b). Regardless of the swab type, it was found that the application of IPA increased the DNA yield compared to dry or water-moistened swabs. The addition of IPA disrupts molecular bonds and precipitates DNA onto the swab, quickly drying it, potentially allowing DNA and cellular components to be more loosely bound to the swab fibres, facilitating their release [35, 36].

### Swab and wetting agent combination selection

Previous research has advocated for the use of a controlled synthetic fingerprint solution in DNA TPPR experiments [43]. The argument being that this solution chemically mimics the deposits left behind during skin contact, and the starting amount and type of DNA within can be controlled and customised. The benefits of such a solution are particularly applicable to DNA recovery, where knowing the amount of starting material is necessary to accurately determine extraction and recovery efficiency. Accordingly, we employed a synthetic touch DNA solution with a defined quantity of starting material, which was tested in parallel with natural touch DNA samples, deposited by volunteers. This allowed us to establish extraction efficiency from known samples and recovery efficiency from deposits with both known and unknown starting amounts, while comparing the behaviour of the synthetic solution to that of natural touch deposits.

While there is no universal agreement on the precise composition of touch DNA deposits, synthetic solutions are designed to include several of the commonly proposed components thought to contribute to touch DNA, including sebaceous secretions [49, 53–57], eccrine secretions [39, 49, 53, 54, 57], cell free DNA [58–63], and cellular DNA [37, 42, 62–65] with these biological constituents being of endogenous or exogenous origin [66]. However, many uncertainties remain regarding the nature of natural touch DNA deposits, hindering the development of synthetic alternatives. Therefore, it should be acknowledged that the synthetic solution currently available for investigative use is neither chemically nor biologically identical to genuine touch DNA, nor does the fluidity of the samples match that of natural touch DNA deposits. Despite these limitations, synthetic solutions provide a closer representation of touch DNA deposits than the currently available viable alternatives (body fluids or buffers). Thus, it is likely that these differences contribute to the differences observed between the two sample types and, consequently, to the variations in rankings of swab and wetting agent combinations between parts one and two. For instance, part one found that a polyester swab moistened with SDS was the first-ranked combination for touch DNA recovery, whereas part two found that a cotton swab moistened with SDS was the first-ranked combination. Additionally, a higher number of statistically significant differences between the combinations was found in part one than in part two of this experiment. In this instance, making a selection of which combination to use for the recovery of natural deposits, based on the results of the synthetic deposits, would result in a suitable selection, as in part two, cotton and SDS do not recover significantly more DNA than the second-place combination polyester and SDS; therefore, the selection based on these data would be sound. Otherwise, if this condition is not met, the swab and wetting agent combination should be selected based on the specific type of DNA being collected (synthetic vs. natural). However, problems arise when determining the order of performance beyond first place. For instance, when observing results from part one, one would assume that polyester and IPA or foam and IPA would be among the top-performing combinations. However, when these combinations are used to collect the natural deposits from part two, they end up 12th and 15th place. Thus, highlighting differences (some of which are statistically significant) in how the synthetic and natural deposits interact with each swab and wetting agent combination.

The high DNA yield of the foam swab wet with IPA in part one was somewhat unexpected. Foam swabs have previously been tested for the collection of touch DNA and identified to increase DNA yield from porous surfaces, such as wood, but reported to have a relatively low recovery efficiency on nonporous surfaces [3, 11]. However, one previous investigation of cellular recovery from fingermarks has reported increased recovery from a non-porous substrate [23]. We hypothesise that the difference in foam swab performance between parts one and two of this experiment may be due to the deposit solutions, in conjunction with the swab’s open structure [21]. The liquid volume of the synthetic trace deposits was 10 µL, which is much greater than would be expected in natural touch DNA samples deposited through hand contact. This may influence the solution’s physicochemical interactions, thereby facilitating increased absorption and adhesion capabilities, potentially altering the interactions between the deposit and the surface and between the deposit and the swab [67–69]. Additionally, although the flat polypropylene sheet was selected because its surface texture resembled that of the 50 mL tube (based on a simple visual and tactile comparison), differences in surface roughness could have affected the swab’s ability to recover DNA from these surfaces [67–69].

Differences among the worst-performing combinations are also evident between the synthetic and natural deposits. When recovering synthetic deposits, swabs moistened with water consistently yielded the lowest DNA amounts, whereas when recovering natural deposits, swabs moistened with IPA yielded the lowest amounts. The amount of sebaceous secretion (lipids) in the synthetic solution, compared with that in the volunteer deposits, could have contributed to the observed differences in DNA recovery between part one and part two. Assuming the lipid content in the synthetic deposits was higher than that deposited by the volunteers (which is a reasonable assumption, as volunteers did not intentionally sebum load their hands prior to contact, and even if they had, deposits would have been expected to be variable), the synthetic deposits would have had increased hydrophobic tendencies, which could have affected the solubility of the DNA, affecting uptake to the swab, resulting in decreased collection. The versatility of the available synthetic fingerprint solutions allows for the easy reduction of sebaceous content in future experiments by diluting the solution in water or synthetic eccrine.

Nonetheless, synthetic fingerprint solutions remain the closest alternative to natural deposits and is currently the only viable method available for standardising touch DNA deposits in studies requiring controlled methods. It is of importance to understand where the differences lie between natural and synthetic deposits, therefore, future research should focus on understanding why synthetic deposits react differently from natural deposits, how to rectify any compositional differences (e.g., amount of lipids to aqueous or biological material), and create amendments to the existing solutions that will allow them to more closely act, not just chemically resemble, natural touch DNA deposits. Therefore, these results demonstrate that volunteer deposits must also be analysed alongside synthetic deposits, regardless of their variable nature, due to the limitations of currently available synthetic solutions.

When considering the study as a whole, it appears that a polyester swab moistened with SDS was one of the most effective combinations for recovering touch DNA. This may have been somewhat unexpected given the overall poor extraction efficiency observed with the polyester swab. However, these findings are consistent with Verdon et al. [3], who also indicate poor extraction efficiency of biological fluids from the MWE polyester swab, yet indicate better recovery efficiency from the same samples. This highlights that recovery efficiency does not equal extraction efficiency and vice versa. Although the polyester and SDS combination ranked second in part two of the experiment, the observed differences between the first and second-ranked combinations were not statistically significant; therefore, it is appropriate to select this swab as it was also the top combination in part one of the study. Irrespective of possible differences in behaviour between natural and synthetic deposits, it appears that polyester and SDS were very high on the list of performance regardless of deposit type. However, with the rest of the swabs, it may be the case that when using synthetic deposits foam and IPA and polyester and IPA are used as second and third options, but in casework, where natural deposits are recovered, cotton and SDS or cotton and IPA would be the most suitable alternatives to polyester and SDS. Our results are consistent with those of Verdon et al. [3], who also found that the MWE polyester swab performed best for recovering touch DNA samples from smooth, non-porous surfaces. However, we also show that cotton had a high recovery efficiency for volunteer-provided touch DNA samples, as reported in the majority of the literature [3, 8, 11, 23]. Conversely, nylon swabs have also been reported to produce high DNA recovery rates when collecting touch DNA [2, 10]; however, these swab types typically ranked in the bottom half of our results (8th-15th), indicating low DNA yield.

In regard to the wetting agents, our results also align with previously published research, indicating that detergents [12–14], such as SDS, or alcohols, such as IPA [5], are among the best moistening agents for maximising DNA recovery. As described by Thomasma and Fredman [14], SDS is amphiphilic, allowing increased solubilisation of cellular components. This means that when using SDS as a wetting agent, the benefits are two-fold: one, increased recovery from the surface, and two, aid in cell lysis during extraction. Thus, this may explain why the recovery efficiency of swabs wet with SDS was high. On the other hand, little is understood about the mechanism by which IPA increases DNA recovery during swabbing. However, the low surface tension of IPA could improve recovery of lipid-rich deposits by avoiding the hydrophobic issues observed with water, as alcohols facilitate the solubilisation of lipids, which could have led to the increased success of IPA with the lipid-rich synthetic deposits. Overall, the results from water-moistened swabs were inconsistent between the two deposit types. Our results show higher DNA yields when using water as a wetting agent during the recovery of natural touch deposits than during the recovery of synthetic touch deposits, a pattern echoed in casework, where water often facilitates successful DNA recovery. Again, this is likely linked to a higher lipid content (sebum) in the synthetic deposits, leading to stronger hydrophobic interactions with this solution, possibly decreasing solubility and resulting in lower recovery. Differences in wetting agent volumes across swab types may have partially affected DNA recovery in some combinations, pushing them towards either the top or the bottom of the rankings. However, the volumes used herein were selected to avoid oversaturating the swab, ensuring no residual liquid remained on the surface after swabbing and thereby decreasing the potential for unrecovered DNA (Supplementary Material 1). Additionally, since only one surface was used in this study, volume effects on the rankings are expected to be reduced, as the rankings are based on data from a single surface type. Furthermore, volume effects have previously been shown to have a greater influence on DNA recovery from porous surfaces and little to no influence on non-porous surfaces; therefore, volume effects on DNA recovery and combination rankings in this study, though possible, are likely minimal [6, 11, 12].

Additionally, differences in the cellular material between the synthetic and natural deposits could have influenced these results. Buccal cells, used as the cellular component of the synthetic deposits, are intact squamous epithelial cells that, in the presence of water (a hypotonic solution), could have lysed, trapping the DNA in the swab matrix, resulting in decreased DNA recovery. On the other hand, natural touch deposits are generally accepted to contain cellular material from shed skin cells that have undergone desquamation, losing their organelles and keratinising. Since these cells are keratinised and lack internal organelles and intact cell walls, they may be more resilient to the hypotonic effects of water. This is in contrast to previous findings, which indicate that water increased the recovery of cellular (mouse embryonic fibroblasts) and cfDNA from synthetic deposits [13], possibly due to the higher sebum content in the synthetic solution used in our study or the difference in cell types. Understanding how varying sebaceous content influences the ability of different wetting agents to recover DNA from a surface would be beneficial, and future research could use synthetic deposits with different percentages of sebaceous secretions to evaluate wetting agent performance. Of note, in instances where water yielded low recovery, water was paired with cotton, viscose, and polyester swabs, which could have provided a complex fibrous matrix, and any protein-bound DNA released through cell lysis could have remained trapped.

Given the observed differences in the behaviour of synthetic and natural deposits, caution should be exercised when applying synthetic solutions beyond controlled studies, such as those focused on method optimisation. This is particularly important for investigations intended to represent different crime scene scenarios for activity-level evaluations, in which it is important to capture the natural variability of touch DNA deposits and accurately reflect the behaviour, chemical and biological elements of real-life conditions. Consequently, extrapolating findings from synthetic deposits to operational casework circumstances must be undertaken with caution and should be reserved for exceptional cases in which no other empirical data exists.

When interpreting these results, it is worth noting that DNA recovery was evaluated using quantification data, and that increased DNA quantity may not correspond to improved STR profile quality. Therefore, the data presented may not fully reflect the potential STR profile completeness, allele recovery, peak height balance, or stoichiometric effects. However, qPCR data provides an inhibition assessment and information on sample degradation, which may help guide expectations for downstream processing and data analysis.

Ensuring that the statistical approach used to analyse the data is appropriate is important for correctly interpreting results when deciding which swab and wetting agent to use for touch DNA collection. As indicated by Verdon et al. [3] and observed by Phetpeng et al. [7], interactions between all variables will influence DNA recovery. Our results concur with these findings, as our statistical analysis shows that the recovery efficiency of touch DNA from a polypropylene surface is strongly influenced by the interactions among the swabs and wetting agents used. Without considering these interactions, it is possible that unwarranted conclusions could be drawn from the data. For instance, without considering the interactions of these variables, based on the results, it would be reasonable to choose IPA or SDS as a wetting agent; however, the choice of swab would be less obvious, and it may be possible that a polyester swab would not be selected; highlighting the importance of considering the effects of all variables on recovery efficiency before selecting a combination for a specific sample type.

## Conclusion

This research aimed to determine the most effective combination of swab and wetting agent for recovering touch DNA from a polypropylene surface using the tested workflow. This research has highlighted that the selection of swabs and wetting agents must be carefully considered, as these combinations can affect the amount of DNA recovered from a surface. Collectively, the results of this study demonstrate that, on a smooth polypropylene surface, polyester swabs moistened with SDS are among the most successful combinations evaluated under the tested conditions. While restricting this investigation to one surface type and extraction kit may seem limiting, it is impractical to test all swabs with all wetting agents on all surfaces with all extraction protocols; therefore, polypropylene was selected based on its abundance in the environment, making it a common surface encountered at crime scenes, and the DNA IQ extraction kit for its common use in forensic casework. Another benefit of selecting polypropylene is that this surface type is often used experimentally to assess DNA transfer and shedder categorisation (e.g., polypropylene 50 mL tubes held by tested individuals) [45–48, 70, 71]. These findings also contribute to our understanding of the factors that influence the recovery of touch DNA, highlighting that DNA recovery protocols should consider the sample properties (type of DNA and the surface on which it is deposited), in addition to the collection system (swab and wetting agent) and extraction as a single workflow, to ensure the recovery protocol is fit for purpose. Thus, this recovery methodology may be adopted for broader uses, such as research or casework, provided the tested conditions remain the same. Therefore, it would be valuable for future research to test additional surfaces of different roughness as well as other common materials to facilitate the determination of appropriate combinations of swabs and wetting agents for each casework circumstance, and laboratory set-up encountered.

## Supplementary Information

Below is the link to the electronic supplementary material.

Supplementary Material 1 (DOCX 6.67 MB)

Supplementary Material 2 (DOCX 9.51 MB)

Supplementary Material 3 (DOCX 8.61 MB)

Supplementary Material 4 (XLSX 84.0 KB)

## Data Availability

All data supporting the findings of this study are available within the paper and its Supplementary Information.

## References

[1] Cook R, Mitchell N, Henry J Assessment of Diamond^™^ Nucleic Acid Dye for the identification and targeted sampling of latent DNA in operational casework. Forensic Sci Int Genet 2021;55. 10.1016/j.fsigen.2021.102579 34455366

[2] Comte J, Baechler S, Gervaix J, Lock E, Milon M-P, Delémont O et al (2019) Touch DNA collection – performance of four different swabs. Forensic Sci Int Genet 43:102113. 10.1016/j.fsigen.2019.06.014 31525724

[3] Verdon TJ, Mitchell RJ, van Oorschot RAH (2014) Swabs as DNA Collection Devices for Sampling Different Biological Materials from Different Substrates. J Forensic Sci 59:1080–1089. 10.1111/1556-4029.12427 24502761

[4] Tozzo P, Mazzobel E, Marcante B, Delicati A, Caenazzo L (2022) Touch DNA sampling methods: efficacy evaluation and systematic review. Int J Mol Sci 23:15541. 10.3390/ijms232415541 PMC9779423 36555182

[5] Bonsu DOM, Higgins D, Henry J, Austin JJ (2021) Evaluation of the efficiency of Isohelix^™^ and Rayon swabs for recovery of DNA from metal surfaces. Forensic Sci Med Pathol 17:199–207. 10.1007/s12024-020-00329-x 33180274

[6] Hedman J, Akel Y, Jansson L, Hedell R, Wallmark N, Forsberg C et al (2021) Enhanced forensic DNA recovery with appropriate swabs and optimized swabbing technique. Forensic Sci Int Genet 53:102491. 10.1016/j.fsigen.2021.102491 33774569

[7] Phetpeng S, Kitpipit T, Thanakiatkrai P (2015) Systematic study for DNA recovery and profiling from common IED substrates: From laboratory to casework. Forensic Sci Int Genet 17:53–60. 10.1016/j.fsigen.2015.03.007 25828367

[8] Comment D, Gouy A, Zingg C, Zieger M (2023) A holistic approach for the selection of forensic DNA swabs. Forensic Sci Int 348:111737. 10.1016/j.forsciint.2023.111737 37247525

[9] Krosch MN, Micic,Biljana N, David H., and, Wilson-Wilde L Preliminary evaluation of three swabs and three wetting agents for the collection of forensic DNA samples from biological fluid stains on a non-porous surface. Aust J Forensic Sci n d ;58:174–184. 10.1080/00450618.2025.2503795

[10] Bruijns BB, Tiggelaar RM, Gardeniers H (2018) The Extraction and Recovery Efficiency of Pure DNA for Different Types of Swabs. J Forensic Sci 63:1492–1499. 10.1111/1556-4029.13837 29890011

[11] Seiberle I, Währer J, Kron S, Flury K, Girardin M, Schocker A et al (2022) Collaborative swab performance comparison and the impact of sampling solution volumes on DNA recovery. Forensic Sci Int Genet 59:102716. 10.1016/j.fsigen.2022.102716 35512614

[12] Schulte J, Rittiner N, Seiberle I, Kron S, Schulz I (2023) Collecting touch DNA from glass surfaces using different sampling solutions and volumes: Immediate and storage effects on genetic STR analysis. J Forensic Sci 68:1133–1147. 10.1111/1556-4029.15305 37282776

[13] Kuffel A, Nic Daeid N, Gray A (2024) Impact of swabbing solutions on the recovery of biological material from non-porous surfaces. Forensic Sci Int Synergy 9:100551. 10.1016/j.fsisyn.2024.100551 PMC11399651 39281425

[14] Thomasma SM, Foran DR (2013) The Influence of Swabbing Solutions on DNA Recovery from Touch Samples. J Forensic Sci 58:465–469. 10.1111/1556-4029.12036 23278347

[15] Oliveira TP, Nogueira TLS, Valentin ESB, Santos OCL, Carvalho EF, Silva DA (2015) Evaluation of collection and extraction methodologies of latent fingerprints for military application. Forensic Sci Int Genet Suppl Ser 5:e474–e475. 10.1016/j.fsigss.2015.09.188

[16] Brownlow RJ, Dagnall KE, Ames CE (2012) A Comparison of DNA Collection and Retrieval from Two Swab Types (Cotton and Nylon Flocked Swab) when Processed Using Three QIAGEN Extraction Methods. J Forensic Sci 57:713–717. 10.1111/j.1556-4029.2011.02022.x 22211626

[17] Dierig L, Schwender M, Wiegand P (2020) Looking for the pinpoint: Optimizing identification, recovery and DNA extraction of micro traces in forensic casework. Forensic Sci Int Genet 44:102191. 10.1016/j.fsigen.2019.102191 31756628

[18] Adamowicz MS, Stasulli DM, Sobestanovich EM, Bille TW (2014) Evaluation of methods to improve the extraction and recovery of DNA from cotton swabs for forensic analysis. PLoS One 9:e116351. 10.1371/journal.pone.0116351 PMC4280208 25549111

[19] Phetpeng S, Kitpipit T, Asavutmangkul V, Duangshatome W, Pongsuwan W, Thanakiatkrai P (2013) Touch DNA collection from improvised explosive devices: A comprehensive study of swabs and moistening agents. Forensic Sci Int Genet Suppl Ser 4:e29–30. 10.1016/j.fsigss.2013.10.014

[20] Conte J, Morgan LA, Johnson TL (2025) Evaluation of swab materials in forensic DNA testing: a systematic review. Egypt J Forensic Sci 15:27. 10.1186/s41935-025-00444-2

[21] Bruijns B (2024) What Are the Limitations and Challenges of Swab-Based DNA Sampling? Forensic Sci 4:76–95. 10.3390/forensicsci4010006

[22] Mulligan CM, Kaufman SR, Quarino L (2011) The Utility of Polyester and Cotton as Swabbing Substrates for the Removal of Cellular Material from Surfaces*. J Forensic Sci 56:485–490. 10.1111/j.1556-4029.2010.01659.x 21342191

[23] Kanokwongnuwut P, Kirkbride P, Linacre A (2018) Visualising latent DNA on swabs. Forensic Sci Int 291:115–123. 10.1016/j.forsciint.2018.08.016 30195152

[24] Vashist V, Banthia N, Kumar S, Agrawal P (2023) A systematic review on materials, design, and manufacturing of swabs. Ann 3D Print Med 9:100092. 10.1016/j.stlm.2022.100092

[25] van Oorschot RA, Ballantyne KN, Mitchell RJ (2010) Forensic trace DNA: a review. Investig Genet 1:14. 10.1186/2041-2223-1-14 PMC3012025 21122102

[26] Sweet D, Lorente M, Lorente JA, Valenzuela A, Villanueva E (1997) An improved method to recover saliva from human skin: the double swab technique. J Forensic Sci. 10.1520/JFS14120J 9068193

[27] Hedman J, Jansson L, Akel Y, Wallmark N, Gutierrez Liljestrand R, Forsberg C et al (2020) The double-swab technique versus single swabs for human DNA recovery from various surfaces. Forensic Sci Int Genet 46:102253. 10.1016/j.fsigen.2020.102253 32007674

[28] Abdullah A, Szkuta B, Meakin GE (2023) Effect of swabbing technique and duration on forensic DNA recovery. Sci Justice. 10.1016/j.scijus.2023.03.002 37169459

[29] Pang BCM, Cheung BKK (2007) Double swab technique for collecting touched evidence. Leg Med 9:181–184. 10.1016/j.legalmed.2006.12.003 17320457

[30] van Oorschot RAH, Phelan DG, Furlong S, Scarfo GM, Holding NL, Cummins MJ (2003) Are you collecting all the available DNA from touched objects? Int Congr Ser 1239:803–807. 10.1016/S0531-5131(02)00498-3

[31] Garvin AM, Holzinger R, Berner F, Krebs W, Hostettler B, Lardi E et al (2013) The forensiX Evidence Collection Tube and Its Impact on DNA Preservation and Recovery. BioMed Res Int 2013:105797. 10.1155/2013/105797 PMC3830855 24288659

[32] Sahayaselvan MD, Chowdhari S, Perumal V, Rastogi S Effects of time, high ambient temperature, swab type, and preservatives on forensic DNA recovery. Sci Justice 2025:101372. 10.1016/j.scijus.2025.101372 41506831

[33] Nolan M, Linacre A (2024) Cell counting to monitor swab efficiency. J Forensic Sci 69:1002–10. 10.1111/1556-4029.15495 38380584

[34] Bonsu DOM, Rodie M, Higgins D, Henry J, Austin JJ (2021) Comparison of Isohelix^™^ and Rayon swabbing systems for touch DNA recovery from metal surfaces. Forensic Sci Med Pathol 17:577–584. 10.1007/s12024-021-00423-8 PMC8528942 34674113

[35] Sophian A (2025) The role of alcohol in the DNA isolation process: A comprehensive review. Kupang J Food Nutr Res 6:47–54

[36] Belotserkovskii BP, Johnston BH (1997) Denaturation and Association of DNA Sequences by Certain Polypropylene Surfaces. Anal Biochem 251:251–262. 10.1006/abio.1997.2249 9299024

[37] Burrill J, Daniel B, Frascione N (2019) A review of trace Touch DNA deposits: Variability factors and an exploration of cellular composition. Forensic Sci Int Genet 39:8–18. 10.1016/j.fsigen.2018.11.019 30513439

[38] Aloraer D, Hassan NH, Albarzinji B, Goodwin W (2017) Improving recovery and stability of touch DNA. Forensic Sci Int Genet Suppl Ser 6:e390–e392. 10.1016/j.fsigss.2017.09.166

[39] Tang J, Ostrander J, Wickenheiser R, Hall A (2020) Touch DNA in forensic science: The use of laboratory-created eccrine fingerprints to quantify DNA loss. Forensic Sci Int Synergy 2:1–16. 10.1016/j.fsisyn.2019.10.004 PMC7219128 32411992

[40] Wood I, Park S, Tooke J, Smith O, Morgan RM, Meakin GE (2017) Efficiencies of recovery and extraction of trace DNA from non-porous surfaces. Forensic Sci Int Genet Suppl Ser 6:e153–e155. 10.1016/j.fsigss.2017.09.022

[41] Bonsu DOM, Higgins D, Austin JJ (2020) Forensic touch DNA recovery from metal surfaces – A review. Sci Justice 60:206–215. 10.1016/j.scijus.2020.01.002 32381237

[42] Oleiwi AA, Morris MR, Schmerer WM, Sutton R (2015) The relative DNA-shedding propensity of the palm and finger surfaces. Sci Justice 55:329–334. 10.1016/j.scijus.2015.04.003 26385715

[43] Arsenault H, Nic Daeid N, Gray A (2023) A synthetic fingerprint solution and its importance in DNA transfer, persistence and recovery studies. Forensic Sci Int Synergy 6:100330. 10.1016/j.fsisyn.2023.100330 PMC10209804 37249970

[44] Polypropylene production worldwide (2026) Statista n.d. https://www.statista.com/statistics/1103529/global-polypropylene-production/?srsltid=AfmBOooW0pfp9MqIkGEo0bztWcr4gecfMrZRqEk45G3hzU4Lgw6A2Smy (accessed February 4, 2026)

[45] Lowe A, Murray C, Whitaker J, Tully G, Gill P (2002) The propensity of individuals to deposit DNA and secondary transfer of low level DNA from individuals to inert surfaces. Forensic Sci Int 129:25–34. 10.1016/S0379-0738(02)00207-4 12230994

[46] Jansson L, Siti C, Hedell R, Forsberg C, Ansell R, Hedman J (2024) Assessing the consistency of shedder status under various experimental conditions. Forensic Sci Int Genet 69:103002. 10.1016/j.fsigen.2023.103002 38176092

[47] Fonneløp AE, Ramse M, Egeland T, Gill P (2017) The implications of shedder status and background DNA on direct and secondary transfer in an attack scenario. Forensic Sci Int Genet 29:48–60. 10.1016/j.fsigen.2017.03.019 28363123

[48] Johannessen H, Gill P, Roseth A, Fonneløp AE (2021) Determination of shedder status: A comparison of two methods involving cell counting in fingerprints and the DNA analysis of handheld tubes. Forensic Sci Int Genet 53:102541. 10.1016/j.fsigen.2021.102541 34090062

[49] Sisco E, Staymates J, Schilling K (2015) A chemically relevant artificial fingerprint material for the cross-comparison of mass spectrometry techniques. Can Soc Forensic Sci J 48:200–214. 10.1080/00085030.2015.1083167

[50] Raymond JJ, van Oorschot RAH, Walsh SJ, Roux C (2008) Trace DNA analysis: Do you know what your neighbour is doing? A multi-jurisdictional survey. Forensic Sci Int Genet 2:19–28. 10.1016/j.fsigen.2007.07.001 19083785

[51] Marshall PL, Stoljarova M, Larue BL, King JL, Budowle B (2014) Evaluation of a novel material, Diomics X-Swab^™^, for collection of DNA. Forensic Sci Int Genet 12:192–198. 10.1016/j.fsigen.2014.05.014 25016249

[52] Evaluation of Swab Materials in Forensic DNA Testing (2026) A Systematic Review | National Institute of Justice n.d. https://nij.ojp.gov/topics/articles/evaluation-swab-materials-forensic-dna-testing-systematic-review

[53] Girod A, Ramotowski R, Weyermann C (2012) Composition of fingermark residue: A qualitative and quantitative review. Forensic Sci Int 223:10–24. 10.1016/j.forsciint.2012.05.018 22727572

[54] Steiner R, Roux C, Moret S (2019) Controlling fingermark variability for research purposes: A review. WIREs Forensic Sci 1:e1338. 10.1002/wfs2.1338

[55] Pleik S, Spengler B, Schäfer T, Urbach D, Luhn S, Kirsch D (2016) Fatty Acid Structure and Degradation Analysis in Fingerprint Residues. J Am Soc Mass Spectrom 27:1565–1574. 10.1007/s13361-016-1429-6 27324649

[56] Croxton RS, Baron MG, Butler D, Kent T, Sears VG (2010) Variation in amino acid and lipid composition of latent fingerprints. Forensic Sci Int 199:93–102. 10.1016/j.forsciint.2010.03.019 20413233

[57] Gm Mong C, Petersen (1999) Trw Clauss. Advanced Fingerprint Analysis Project Fingerprint Constituents. 10.2172/14172

[58] Kita T, Yamaguchi H, Yokoyama M, Tanaka T, Tanaka N (2008) Morphological study of fragmented DNA on touched objects. Forensic Sci Int Genet 3:32–36. 10.1016/j.fsigen.2008.09.002 19083864

[59] Quinones I, Daniel B (2012) Cell free DNA as a component of forensic evidence recovered from touched surfaces. Forensic Sci Int Genet 6:26–30. 10.1016/j.fsigen.2011.01.004 21292581

[60] Burrill J, Kombara A, Daniel B, Frascione N (2021) Exploration of cell-free DNA (cfDNA) recovery for touch deposits. Forensic Sci Int Genet 51:102431. 10.1016/j.fsigen.2020.102431 33260058

[61] Vandewoestyne M, Van Hoofstat D, Franssen A, Van Nieuwerburgh F, Deforce D (2013) Presence and potential of cell free DNA in different types of forensic samples. Forensic Sci Int Genet 7:316–320. 10.1016/j.fsigen.2012.12.005 23318134

[62] Stanciu CE, Philpott MK, Kwon YJ, Bustamante EE, Ehrhardt CJ Optical characterization of epidermal cells and their relationship to DNA recovery from touch samples. F1000Research 2015;4. 10.12688/f1000research.7385.1 PMC4732551 26870321

[63] Miller M, Philpott MK, Olsen A, Tootham M, Yadavalli VK, Ehrhardt CJ (2021) Technical note: Survey of extracellular and cell-pellet-associated DNA from ‘touch’/trace samples. Forensic Sci Int 318:110557. 10.1016/j.forsciint.2020.110557 33168421

[64] Balogh MK, Burger J, Bender K, Schneider PM, Alt KW (2003) Fingerprints from fingerprints. Int Congr Ser 1239:953–957

[65] Recipon M, Agniel R, Kunemann P, Ponche A, Carreiras F, Hermitte F et al (2024) Detection of invisible biological traces in relation to the physicochemical properties of substrates surfaces in forensic casework. Sci Rep 14:13271. 10.1038/s41598-024-63911-1 PMC11164948 38858407

[66] Burrill J, Hotta R, Daniel B, Frascione N (2021) Accumulation of endogenous and exogenous nucleic acids in Touch DNA components on hands. Electrophoresis 42:1594–1604. 10.1002/elps.202000371 34080688

[67] Hughes DA, van Oorschot RAH, Szkuta B, Conlan XA (2024) DNA recovery and substrate considerations; more than a superficial relationship. Aust J Forensic Sci 56:178–180. 10.1080/00450618.2024.2324788

[68] Hughes DA, Szkuta B, van Oorschot RAH, Conlan XA (2024) The impact of substrate characteristics on the collection and persistence of biological materials, and their implications for forensic casework. Forensic Sci Int 356:111951. 10.1016/j.forsciint.2024.111951 38301431

[69] Hughes DA, Szkuta B, van Oorschot RAH, Yang W, Conlan XA (2021) Impact of surface roughness on the deposition of saliva and fingerprint residue on non-porous substrates. Forensic Chem 23:100318. 10.1016/j.forc.2021.100318

[70] Ali D, van Oorschot RAH, Linacre A, Goray M (2024) How to best assess shedder status: a comparison of popular shedder tests. Int J Legal Med. 10.1007/s00414-024-03351-8 PMC12003581 39508835

[71] Jansson L, Swensson M, Gifvars E, Hedell R, Forsberg C, Ansell R et al (2022) Individual shedder status and the origin of touch DNA. Forensic Sci Int Genet 56:102626. 10.1016/j.fsigen.2021.102626 34781198

