# Supplementary material for: A swab and wetting agent combination investigation for enhanced touch DNA recovery: A step towards standardisation
Source: Int J Legal Med. 2026 Jul 4;140(6):4135–56. doi: 10.1007/s00414-026-03907-w (PMC13658405; doi:10.1007/s00414-026-03907-w)
Supplement: Supplementary file 1 [file 414_2026_3907_MOESM1_ESM.docx]

**Title:** A swab and wetting agent combination investigation for enhanced touch DNA recovery: A step towards standardisation

**Journal:** International Journal of Legal Medicine

**Authors:** Hilary Arsenault^1*^, Roland A.H. van Oorschot^2,3^, Duncan Taylor^1,4^, Adrian Linacre^1^, Mariya Goray^1^

**Affiliations**

^1^College of Science and Engineering, Flinders University, Adelaide, SA, 5042, Australia

^2^ Office of the Chief Forensic Scientist, Victoria Police Forensic Services, Macleod, Australia

^3^School of Agriculture, Biomedicine and Environment, La Trobe University, Bundoora, Australia

^4^Forensic Science SA, Adelaide, SA 5000, Australia

*Corresponding author: [hilary.arsenault@flinders.edu.au](mailto:hilary.arsenault@flinders.edu.au)

## **Online Resource 1: Preliminary experiments**

1.1 Buccal cell re-suspension and cell integrity

Buccal cells were used as the cellular fraction of the synthetic touch DNA deposit solution used in part one of this experiment. However, since the aim was to have a deposit solution that closely mimicked touch DNA deposits, saliva was not an acceptable deposit medium for the DNA. This required the buccal cells to be separated from the saliva and prepared in a manner that allowed them to be added to a deposit master mix. Therefore, the buccal cells were resuspended following a standard molecular biology technique [1–4], outlined briefly in the Materials and methods, section 2.2.1.

To ensure the buccal cells maintained their integrity during the resuspension process, two test resuspensions were performed. One aliquot was resuspended in 200 μL 1x PBS, and the other in 200 μL of a synthetic fingerprint solution (BioChemazone, CA). Three 10 μL aliquots of each of the two cell resuspensions were deposited on a glass microscope slide and allowed to dry. Negative controls of the synthetic fingerprint solution and 1x PBS were also obtained. Samples were then visualised using Diamond^TM^ Nucleic Acid dye (DD) (Promega, Madison, USA) and a microscope (as outlined in detail in Materials and methods, section 2.2.1), allowing cell integrity (intact or damaged) to be determined.

During testing of the resuspension methodology, it was found that one centrifugation step was insufficient to remove all salivary secretions, as the 200 μL resuspension (intended for addition to the master mix) still contained saliva (the solution remained viscous and sticky). Therefore, a wash step with 1,000 μL of 1x PBS was introduced between the first centrifugation and the final resuspension. This step successfully removed all salivary secretions from the pellet, allowing it to be resuspended properly. During this testing, it was also found that centrifugation at 10,000 g was necessary, as lower speeds did not yield a well-defined cell pellet, as observed when the cells were disrupted by the flow of the supernatant during removal.

The results of this testing show that after resuspension, the buccal cells’ cell walls and nuclei were intact and comparable to those found in neat saliva, as shown in Figure S1.1. Therefore, this cell resuspension method was deemed adequate for removing buccal cells from saliva for use as the cellular component of the synthetic touch DNA deposit solution. Negative controls with 1x PBS and the synthetic fingerprint solution showed no interference from the solutions on cell visualisation (Fig. S1.1).

**Fig S1.1**: Buccal cells after resuspension. a) Neat saliva, b) buccal cells resuspended in 1X PBS, c) buccal cells resuspended in the synthetic fingerprint material, d) negative synthetic fingerprint solution, and e) negative 1X PBS. Images A1 to E1 are magnified 220x, and images A2 to E2 are close-ups of the cells present at 220x magnification

1.2 Wetting agents: Deposition method

Prior to the application of wetting agents to the test swabs, the devices used for application were tested to determine the most effective wetting method. In an effort to standardise the swab method and eliminate unnecessary variability, two commonly used devices for wetting agent application were tested to determine whether they consistently delivered the same volume of solution. Therefore, we tested the 10 mL prefilled water ampule (water for injection BP, Medisa, Australia) in delivering the same sized drops by depositing five replicates of 1 and 3 drops of water into 1.5 mL tubes. Afterwards, the volume of liquid was measured using a micropipette, and the resultant volumes were recorded. This was then repeated with three drops of water dispensed from a Pasteur pipette.

Results from the deposition instrument testing show that neither device consistently delivered drops of the same volume (Table S1.1). To minimise variability in the volume of the applied wetting agent, a micropipette was deemed most appropriate for depositing the wetting agent onto the swab in the main experiment, as this device could be relied upon to deliver consistent volumes of solution. Additionally, this mode of wetting agent deposition avoided issues associated with finding ampules suitable for dispensing the other wetting agents, as these were not pre-packaged in ampules like water.

**Table S1.1** Recorded drop volumes produced by the ampule and Pasture pipette

| Wetting agent deposition device | Number of drops | Replicate | Volume (ul) |
| --- | --- | --- | --- |
| 10 mL pre-filled water ampule | 1 | 1 | 58 |
|  | 1 | 2 | 66 |
|  | 1 | 3 | 69 |
|  | 1 | 4 | 66 |
|  | 1 | 5 | 62 |
| 10 mL pre-filled water ampule | 3 | 1 | 160 |
|  | 3 | 2 | 184 |
|  | 3 | 3 | 250 |
|  | 3 | 4 | 200 |
|  | 3 | 5 | 226 |
| Pasture pipette | 1 | 1 | 136 |
|  | 1 | 2 | 121 |
|  | 1 | 3 | 130 |
|  | 1 | 4 | 117 |
|  | 1 | 5 | 124 |

1.3 Wetting agents: Volumes

The optimal volume of wetting agent to apply to each swab, ensuring the swab was sufficiently saturated for swabbing without oversaturation, was tested. In this case, a swab was considered oversaturated when it either could not absorb all the water applied, causing it to drip from the swab after application, or could not collect all the moisture from a surface after swabbing. One and three drops of water from the 10 ml pre-filled ampule were deposited onto each test swab, in addition to volumes of 10 μL, 20 μL, 50 μL, and 100 μL dispensed using a micropipette. These volumes were selected based on previously published literature [5–10] and observations from the application device tests (Online Resource 1, section 1.2). Swabs were visually inspected for signs of oversaturation (dripping) after water application, and the interaction of the water with the swab was recorded. These swabs were then used to swab a blank deposit surface (surface specifics and swab protocol can be found in Online Resource 2, sections 2.2 and 2.3), and the moisture level remaining on the surface after swabbing was visually assessed and recorded.

The results of the wetting agent volume tests showed that the volume required to moisten a swab head without oversaturation varied by swab type. Table S1.2 shows the observations made after each volume of water was applied to each swab type and after a test swab of a flat 3.5 cm by 3.5 cm polypropylene surface was conducted.

**Table S1.2** Observations of absorption capacity of swabs-based water application

| Swab | Volume | Observations |
| --- | --- | --- |
| Viscose  (Sarstedt) | 1 drop | Mild oversaturation   - One replicate liquid hanging from the swab but not dropping - One replicate shows insufficient moisture absorption from the surface after swabbing |
|  | 3 drops | Oversaturated   - Dripping from the swab after application - Insufficient moisture absorption from the surface after swabbing |
|  | 10 ul | Undersaturated   - The majority of the swab was dry - Insufficient moisture for surface swabbing |
|  | 20 ul | Undersaturated   - The majority of the swab was dry - Insufficient moisture for surface swabbing |
|  | 50 ul | Absorbed   - All liquid absorbed by the swab - Sufficient mister absorption from the surface after swabbing |
|  | 100 ul | Oversaturated   - Insufficient moisture absorption from the surface after swabbing |
| Cotton  (Puritan) | 1 drop | Mild oversaturation   - One replicate liquid hanging from the swab but not dropping - One replicate showed insufficient moisture absorption from the surface after swabbing |
|  | 3 drops | Oversaturated   - Dripping from the swab after application - Insufficient moisture absorption from the surface after swabbing |
|  | 10 ul | Undersaturated   - The majority of the swab was dry - Insufficient moisture for surface swabbing |
|  | 20 ul | Undersaturated   - The majority of the swab was dry - Insufficient moisture for surface swabbing |
|  | 50 ul | Absorbed   - All liquid absorbed by the swab - Sufficient mister absorption from the surface after swabbing |
|  | 100 ul | Mild oversaturated   - All liquid absorbed by the swab - Insufficient moisture absorption from the surface after swabbing |
| Nylon  (Copan) | 1 drop | Dry   - Water beaded up and fell from the swab - Remained as a droplet on the swab surface |
|  | 3 drops | Dry   - Water beaded up and fell from the swab - Remained as a droplet on the swab surface |
|  | 10 ul | Absorbed   - All liquid absorbed by the swab - Sufficient mister absorption from the surface after swabbing |
|  | 20 ul | Mild oversaturated   - All liquid absorbed by the swab - Insufficient moisture absorption from the surface after swabbing |
|  | 50 ul | Mild oversaturated   - All liquid absorbed by the swab - Insufficient moisture absorption from the surface after swabbing |
|  | 100 ul | Oversaturated   - Dripping from the swab after application - Insufficient moisture absorption from the surface after swabbing |
| Foam  (Puritan) | 1 drop | Dry   - Water beaded up and fell from the swab - Remained as a droplet on the swab surface |
|  | 3 drops | Dry   - Water beaded up and fell from the swab - Remained as a droplet on the swab surface |
|  | 10 ul | Absorbed   - All liquid absorbed by the swab - Sufficient mister absorption from the surface after swabbing |
|  | 20 ul | Mild oversaturated   - All liquid absorbed by the swab - Insufficient moisture absorption from the surface after swabbing |
|  | 50 ul | Mild oversaturated   - All liquid absorbed by the swab - Insufficient moisture absorption from the surface after swabbing |
|  | 100 ul | Oversaturated   - Dripping from the swab after application - Insufficient moisture absorption from the surface after swabbing |
| Polyester  (MWE) | 1 drop | Mild oversaturation   - All liquid absorbed by the swab - Insufficient moisture absorption from the surface after swabbing |
|  | 3 drops | Oversaturated   - Dripping from the swab after application - Insufficient moisture absorption from the surface after swabbing |
|  | 10 ul | Undersaturated   - The majority of the swab was dry - Insufficient moisture for surface swabbing |
|  | 20 ul | Undersaturated   - The majority of the swab was dry - Insufficient moisture for surface swabbing |
|  | 50 ul | Absorbed   - All liquid absorbed by the swab - Sufficient mister absorption from the surface after swabbing |
|  | 100 ul | Mild oversaturated   - All liquid absorbed by the swab - Insufficient moisture absorption from the surface after swabbing |

Based on the results of this preliminary test, 50 μL of wetting agent was selected for use with viscose, cotton and polyester swabs and 10 μL for nylon and foam swabs. Since nylon and foam swabs exhibit hydrophobic tendencies during the application of water, absorption was aided by lightly contacting and rubbing the pipette tip against the swab head during application. This contact encouraged water absorption into the swab. To confirm that these volumes would be suitable for all wetting agents, each swab was tested once with the designated volume, and the remaining two wetting agents (2% SDS and 100% IPA). The results of this test confirm that the volumes identified in the water test are sufficient for use with all wetting agents. Additionally, observations reveal that nylon and foam swabs do not exhibit the same hydrophobic tendencies with these wetting agents as they did with water.

## References:

[1]	Helgason CD. Culture of Primary Adherent Cells and a Continuously Growing Nonadherent Cell Line n.d.

[2]	Brooks PJ, Malkin EZ, Michino SD, Bratman SV. Isolation of salivary cell-free DNA for cancer detection. PLOS ONE 2023;18:e0285214. https://doi.org/10.1371/journal.pone.0285214.

[3]	Lim Y, Totsika M, Morrison M, Punyadeera C. The saliva microbiome profiles are minimally affected by collection method or DNA extraction protocols. Sci Rep 2017;7:8523. https://doi.org/10.1038/s41598-017-07885-3.

[4]	Zeringer E, Li M, Barta T, Schageman J, Pedersen KW, Neurauter A, et al. Methods for the extraction and RNA profiling of exosomes. World J Methodol 2013;3:11–8. https://doi.org/10.5662/wjm.v3.i1.11.

[5]	Bruijns BB, Tiggelaar RM, Gardeniers H. The Extraction and Recovery Efficiency of Pure DNA for Different Types of Swabs. J Forensic Sci 2018;63:1492–9. https://doi.org/10.1111/1556-4029.13837.

[6]	Schulte J, Rittiner N, Seiberle I, Kron S, Schulz I. Collecting touch DNA from glass surfaces using different sampling solutions and volumes: Immediate and storage effects on genetic STR analysis. J Forensic Sci 2023;68:1133–47. https://doi.org/10.1111/1556-4029.15305.

[7]	Abdullah A, Szkuta B, Meakin GE. Effect of swabbing technique and duration on forensic DNA recovery. Sci Justice 2023. https://doi.org/10.1016/j.scijus.2023.03.002.

[8]	Hedman J, Akel Y, Jansson L, Hedell R, Wallmark N, Forsberg C, et al. Enhanced forensic DNA recovery with appropriate swabs and optimized swabbing technique. Forensic Sci Int Genet 2021;53:102491. https://doi.org/10.1016/j.fsigen.2021.102491.

[9]	Seiberle I, Währer J, Kron S, Flury K, Girardin M, Schocker A, et al. Collaborative swab performance comparison and the impact of sampling solution volumes on DNA recovery. Forensic Sci Int Genet 2022;59:102716. https://doi.org/10.1016/j.fsigen.2022.102716.

[10] Brownlow RJ, Dagnall KE, Ames CE. A Comparison of DNA Collection and Retrieval from Two Swab Types (Cotton and Nylon Flocked Swab) when Processed Using Three QIAGEN Extraction Methods. J Forensic Sci 2012;57:713–7. https://doi.org/10.1111/j.1556-4029.2011.02022.x.
