# Supplementary material for: A swab and wetting agent combination investigation for enhanced touch DNA recovery: A step towards standardisation
Source: Int J Legal Med. 2026 Jul 4;140(6):4135–56. doi: 10.1007/s00414-026-03907-w (PMC13658405; doi:10.1007/s00414-026-03907-w)
Supplement: Supplementary file 2 [file 414_2026_3907_MOESM2_ESM.docx]

**Title:** A swab and wetting agent combination investigation for enhanced touch DNA recovery: A step towards standardisation

**Journal:** International Journal of Legal Medicine

**Authors:** Hilary Arsenault^1*^, Roland A.H. van Oorschot^2,3^, Duncan Taylor^1,4^, Adrian Linacre^1^, Mariya Goray^1^

**Affiliations**

^1^College of Science and Engineering, Flinders University, Adelaide, SA, 5042, Australia

^2^ Office of the Chief Forensic Scientist, Victoria Police Forensic Services, Macleod, Australia

^3^School of Agriculture, Biomedicine and Environment, La Trobe University, Bundoora, Australia

^4^Forensic Science SA, Adelaide, SA 5000, Australia

*Corresponding author: [hilary.arsenault@flinders.edu.au](mailto:hilary.arsenault@flinders.edu.au)

## **Online Resource 2: Experimental Methodology**

2.1 Part one: Surface cleaning

To create DNA-free surfaces, the polypropylene sheets were cleaned. During initial cleaning of the sheets using the standard 1% sodium hypochlorite solution, deionised water and 70% ethanol, it was found that paper towel residue remained, adhering to the sheets surface. To avoid this, both sides of the sheets were exposed to UV irradiation (BLX Bio-Link Crosslinker, Vilber) for 30 minutes as an alternative method of cleaning. After UV exposure, it was observed that the polypropylene had changed colour, turning opaque yellow from its original translucent clear (Fig. S2.1). It was hypothesised that this colour change was due to photooxidation, a process that degrades plastic by causing breaks in long polymer chains [1,2]. This process can cause the formation of structures such as chromophores, leading to yellowing [1,2]. Because this process degrades the polypropylene sheet, an alternative cleaning regimen was needed. Results showed that a more durable wipe (Teri Wipes) did not deteriorate on the surface during the 1% sodium hypochlorite solution, deionised water and 70% ethanol method; therefore, this approach was determined to be best for surface cleaning.

**Fig. S2.1** Polypropylene sheet used for deposits in part one. a) Before UV irradiation, b) after UV irradiation.

2.2 Part one: Deposit surface

**Fig. S2.2** Part one DNA deposit surface. The polypropylene deposit surface features a 3x5 grid, consisting of an inner 3.5 cm x 3.5 cm square attached to the underside. Five surfaces were made, one for each swab type. Each column indicates samples to be collected with a different wetting agent

2.3 Part one: Swabbing protocol

Swabs were moistened with the designated volume of wetting agent, as determined in Online Resource 1, then placed at the top left corner of the swab square at a 45° angle. While rotating, the swab was moved in a zigzag pattern across the entire area of the 3.5 cm by 3.5 cm square (Fig. S2.3, a) in both forward and backward directions (Fig. S2.3, b). This was repeated a total of 4 times following the directions indicated in Fig. S2.3, b.

**Fig. S2.3** Swab method used to collect synthetic touch DNA deposits from a flat polypropylene sheet. a) Zig-zag pattern swab was moved along the surface in, b) red arrow indicates the first direction swabbed, green arrow indicates the second direction swabbed, black arrow indicates the third direction swabbed, purple arrow indicates the fourth direction swabbed

2.4 Part two: Volunteer protocol for DNA deposition

The prescribed handling procedure was as follows: 1) using their right-hand, grip the top suspended tube with the palm facing down, twist the hand once forward around the circumference of the tube and then back (Fig. S2.4, b), 2) change the grip so that the palm is now facing up and twist the hand once forward around the circumference of the tube and then back (Fig. S2.4, c), 3) change grip and hold the tube with the palm facing down, twist the hand backwards and forwards around the circumference of the tube while traveling down the length of the tube and back (Fig. S2.4, d) 4) change grip and hold the tube with the palm facing up, twist the hand backwards and forwards around the circumference of the tube while traveling down the length of the tube and back (Fig. S2.4, e). Once this prescribed action was completed with the right hand, it was repeated on the second tube (the bottom suspended tube) with the left hand (Fig. S2.4, a).

**Fig. S2.4** Part two: DNA deposition apparatus. a) retort stand suspending two 50 mL tubes ready for DNA deposition. The top tube was designated for deposits from the right hand, and the bottom tube for deposits from the left hand. b-e) tube handling method for DNA deposition, where the orange arrow indicates forward around the circumference of the tube, the blue arrow indicates backward around the circumference of the tube, the green arrow indicates travel lengthwise down the tube, and the pink arrow indicates travel lengthwise up the tube

2.5 Part two: Swabbing protocol

The 50 mL tubes were swabbed in a controlled and standardised manner. As previously indicated, swab guides were inserted into the tubes, creating four equally sized collection quarters. Half of the tube (green and purple quarters) was designated for DNA collection with the test swab, and the other half (orange and blue) was designated for DNA collection with the control swab (Fig. S2.5). After moistening the test swab, it was placed on the surface of the 50 mL tube at a 45° angle, at the start point (Fig. S2.5, c). The swab was rotated and moved in a zig-zag pattern across half of the tube designated for collection with the test swab (Fig. S2.5, a), following the forward and backward directions as indicated by the purple and orange arrows in Fig. S2.5, c. A second pass with the test swab of the corresponding half of the tubes' surface was performed, but this time both quarters (making up the half) of the designated swab area were swabbed separately (Fig. S2.5, b (green and purple)), following the forward and backward directions (Fig. S2.5, c). Upon completion, the test swabs were returned to their labelled container for drying. This process was then repeated on the control section of the same tube using the control swab and corresponding wetting agent. Control swabs were returned to their labelled container for drying, and all swabs were stored in paper envelopes until DNA extraction (Materials and methods, section 2.7).

**Fig. S2.5** Swab method used to collect volunteer-deposited touch DNA from a 50 mL tube. Coloured swab guides indicate a) the first swab step where the entire section was swabbed, red arrows indicate the zig-zag movement, b) the second swab step where the individual sections were swabbed, red arrows indicate the zig-zag movement, and c) the direction of swabbing, where the green line denotes the start and stop point, purple arrows indicate the forward direction, and orange arrows indicate the backward direction. A black X indicates an area of the tube that was not swabbed

2.6 Swabs

**Fig. S2.6** Swabs used in this experiment with their packaging. a) Sarstedt, viscose; b) Puritan, Cotton; c) Copan, nylon; d) Puritan, Foam; and e) MWE, polyester

## References:

[1]	Doğan M. Ultraviolet light accelerates the degradation of polyethylene plastics. Microsc Res Tech 2021;84:2774–83. https://doi.org/10.1002/jemt.23838.

[2]	Yousif E, Haddad R. Photodegradation and photostabilization of polymers, especially polystyrene: review. SpringerPlus 2013;2:398. https://doi.org/10.1186/2193-1801-2-398.
