# Supplementary material for: A swab and wetting agent combination investigation for enhanced touch DNA recovery: A step towards standardisation
Source: Int J Legal Med. 2026 Jul 4;140(6):4135–56. doi: 10.1007/s00414-026-03907-w (PMC13658405; doi:10.1007/s00414-026-03907-w)
Supplement: Supplementary file 3 [file 414_2026_3907_MOESM3_ESM.docx]

**Title:** A swab and wetting agent combination investigation for enhanced touch DNA recovery: A step towards standardisation

**Journal:** International Journal of Legal Medicine

**Authors:** Hilary Arsenault^1*^, Roland A.H. van Oorschot^2,3^, Duncan Taylor^1,4^, Adrian Linacre^1^, Mariya Goray^1^

**Affiliations**

^1^College of Science and Engineering, Flinders University, Adelaide, SA, 5042, Australia

^2^ Office of the Chief Forensic Scientist, Victoria Police Forensic Services, Macleod, Australia

^3^School of Agriculture, Biomedicine and Environment, La Trobe University, Bundoora, Australia

^4^Forensic Science SA, Adelaide, SA 5000, Australia

*Corresponding author: [hilary.arsenault@flinders.edu.au](mailto:hilary.arsenault@flinders.edu.au)

**Online Resource 3: Experimental Results**

3.1 General observations: Swab type and wetting agent

During sample collection, general observations were recorded regarding the ease of use of the swabs and wetting agents, which are presented in Table S3.1. The noteworthy observations regarding the use of the swabs, as assessed by the researcher, are as follows. The nylon swab was the most comfortable to handle, whereas the cotton swab was the least comfortable. The polyester swab was the most difficult to handle due to its weak shaft and was the only type to break during swabbing. Nylon and foam swabs showed hydrophobic tendencies when water was used as a wetting agent. Water either remained in a droplet on the surface of these swabs without soaking in or beaded up and fell from the swab. These tendencies were not observed when using the other wetting agents. Polyester and viscose exhibited delayed absorption tendencies during water deposition, requiring a slight increase in time to absorb. Regarding head size, cotton swabs were the only swabs that exhibited visually discernible differences in head size among the swabs tested.

Regarding the observations on wetting agents, all were readily available and relatively low-cost. Both water and IPA could be used as purchased, but SDS required subsequent dilution to 2% prior to use as a wetting agent. When considering recovery from a surface, water did not readily absorb back into foam, nylon, or polyester swabs. Collection with SDS created small bubbles on the collection surface, requiring an additional light pass with the swab after the prescribed swab method to ensure that all liquid (and bubbles) were absorbed back into the swab. IPA evaporated quickly from the swab and the surface, and in some cases, also removed small amounts of blue ink from the outer surface of 50 mL tubes. Additionally, when using IPA as a wetting agent with polyester and viscose swabs, streaks were visible on the surface after swabbing was complete. These streaks were assumed to be glue from the swab heads or residue from the sebaceous portion of the deposits.

Observations were also documented during the extraction process. The primary observation noted during extraction was that with polyester swabs, some samples contained an unknown white substance, thought to be a precipitate [1] or swab head fibres. Additionally, in some samples, the magnetic resin did not pellet as tightly as expected, resulting in a diffused looking pellet. Regarding swabs wet with IPA that exhibited blue discolouration and the sample moistened with SDS exhibiting possible precipitate, these conditions appeared to have no adverse effects on the extraction process and no inhibition during quantification [1,2].

**Table S3.1** General observations made during swab and wetting agent testing. Where numeric indicators are used, 1 represents the best performance, 2 represents medium performance, and 3 represents the poorest performance

| Abbreviation | Brand | Handle stability | Swab head integrity^1^ | Ease of insertion back into container | Head breaks from the shaft | Drying system | Wetting agent volume (µL) | Water absorption | IPA 100%  absorption | SDS 2%  absorption | Cost per swab | Delivery^2^ | Communication^2^ |
| --- | --- | --- | --- | --- | --- | --- | --- | --- | --- | --- | --- | --- | --- |
| Viscose | Sarstedt | Sturdy | 1 | 1 | 1 | Membrane | 50 | 1 | 1 | 1 | Low | Fast | Good |
| Cotton | Puritan Medical | Flimsy | 2 | 2 | 1 | Filter | 50 | 1 | 1 | 1 | High | Fast | Good |
| Nylon | Copan | Extremely sturdy | 1 | 1 | 1 | No built in system, manual aeration needed | 10 | 3 | 1 | 1 | Low | Slow | Good |
| Foam | Puritan Medical | Sturdy | 2 | 1 | 1 | No built in system, manual aeration needed | 10 | 3 | 1 | 1 | Low | Fast | Good |
| Polyester | Medical Wire (MWE) | Very flimsy | 2 | 2 | 1 | No built in system, manual aeration needed | 50 | 2 | 1 | 1 | Very Low | Very Slow | Poor |

^1^This section refers to the ability of the swab head to stay tightly bound (intact) during swabbing

^2^This section is based on the purchase experience of our lab. Circumstances may vary depending on the laboratory location and stock availability. The possibility of temporary, unfortunate circumstances could have led to this outcome

3.2 Part one: Cell integrity: Microscopic examination of the synthetic touch DNA deposit solution used for collection with viscose, cotton, nylon and foam swabs

Microscopic examination of the synthetic touch DNA deposit solution confirms that buccal cells, used as the cellular fraction of the controlled deposition solution, sustained no damage during resuspension. Fig. S3.2 shows that the cells in the deposit solution exhibit the same cell structure (intact cell walls and nuclei) as those from neat saliva.

**Fig. S3.2** Buccal cells viewed under fluorescent microscopy at 220X magnification. a) Neat saliva, b) synthetic touch DNA master mix deposited immediately after being made, c) synthetic touch DNA master mix deposited immediately prior to the halfway point of sample deposition, d) synthetic touch DNA master mix deposited immediately after the halfway point of sample deposition, and e) synthetic touch DNA master mix deposited after all depositions have been made. These images are from the deposit solution used for collection with viscose, cotton, nylon and foam swab

3.3 Part one: Cell integrity: Microscopic examination of the synthetic touch DNA deposit solution used for collection with polyester swabs

Due to a delay in receiving the polyester swabs, two synthetic touch DNA deposit solutions were made. Fig. S3.3 shows that the cells used in the deposit for samples collected with polyester swabs exhibited the same cell structure (intact cell walls and nuclei) as those from neat saliva.

**Fig. S3.3** Buccal cells viewed under fluorescent microscope at 220X magnification. a) Neat saliva, b) synthetic touch DNA master mix deposited immediately after being made, c) synthetic touch DNA master mix deposited halfway through sample deposition, d) synthetic touch DNA master mix deposited after all depositions have been made. These images are from the deposit solution used for collection with polyester swabs

3.3.1 Part one: Cell clumping

Despite following proper mixing and resuspension protocols, cellular material is susceptible to clumping. Therefore, any solution containing cellular material contains a small amount of inherent variability (Fig. S3.4). This can pose issues for DNA analysis in samples containing cell clumps. Therefore, anomalies observed when using synthetic deposits may be explained by cell clumps or the inherent nature of synthetic deposits. Future work should focus on overcoming cell clumping to eliminate outliers and ensure consistent, uniform deposits while maintaining chemical relevance to natural deposits.

**Fig. S3.4** Close-up of a buccal cell clump. Sample of the synthetic touch DNA deposit solution used for sample deposition. Cells were viewed under a fluorescent microscope at 220X magnification

References

[1]	Schulte J, Rittiner N, Seiberle I, Kron S, Schulz I. Collecting touch DNA from glass surfaces using different sampling solutions and volumes: Immediate and storage effects on genetic STR analysis. J Forensic Sci 2023;68:1133–47. https://doi.org/10.1111/1556-4029.15305.

[2]	DNA IQ^TM^ System n.d. https://www.promega.com.au/products/forensic-dna-analysis-ce/dna-isolation/dna-iq-system/ (accessed January 13, 2026).
